# Microbial and metabolic characterization of organic artisanal sauerkraut fermentation and study of gut health-promoting properties of sauerkraut brine

**DOI:** 10.3389/fmicb.2022.929738

**Published:** 2022-10-13

**Authors:** Giulia Gaudioso, Tobias Weil, Giulia Marzorati, Pavel Solovyev, Luana Bontempo, Elena Franciosi, Luigi Bertoldi, Carlo Pedrolli, Kieran Michael Tuohy, Francesca Fava

**Affiliations:** ^1^Nutrition and Nutrigenomics Unit, Research and Innovation Center, Fondazione Edmund Mach, San Michele all'Adige, Italy; ^2^Centre for Integrative Biology (CIBIO) – Department of Cellular, Computational and Integrative Biology, University of Trento, Trento, Italy; ^3^Traceability Unit, Research and Innovation Center, Fondazione Edmund Mach, San Michele all'Adige, Italy; ^4^Organic Agriculture Unit, Environmental Department, Technology Transfer Center, Fondazione Edmund Mach, San Michele all'Adige, Italy; ^5^Dietetics and Clinical Nutrition, Nutrition Department, S. Chiara Hospital, Azienda Provinciale per I Servizi Sanitari, Trento, Italy; ^6^School of Food Science and Nutrition, University of Leeds, Leeds, United Kingdom

**Keywords:** fermented food, sauerkraut, lactic acid bacteria, food microbiota, gut barrier, microbial metabolites

## Abstract

Sauerkraut is a traditionally fermented cabbage, and recent evidence suggests that it has beneficial properties for human health. In this work, a multi-disciplinary approach was employed to characterize the fermentation process and gut health-promoting properties of locally produced, organic sauerkraut from two distinct producers, SK1 and SK2. 16S rRNA metataxonomics showed that bacterial diversity gradually decreased as fermentation progressed. Differences in sauerkraut microbiota composition were observed between the two producers, especially at the start of fermentation. Lactic acid bacteria (LAB) dominated the microbiota after 35 days, with *Lactiplantibacillus* being the dominant genus in both sauerkraut products, together with *Leuconostoc* and *Paucilactobacillus* in SK1, and with *Pediococcus, Levilactibacillus*, and *Leuconostoc* in SK2. LAB reached between 7 and 8 Log CFU/mL brine at the end of fermentation (35 days), while pH lowering happened within the first week of fermentation. A total of 220 LAB strains, corresponding to 133 RAPD-PCR biotypes, were successfully isolated. *Lactiplantibacillus plantarum* and *Lactiplantibacillus pentosus* accounted for 67% of all SK1 isolates, and *Lactiplantibacillus plantarum/paraplantarum* and *Leuconostoc mesenteroides* represented 72% of all the isolates from SK2. ^1^H-NMR analysis revealed significant changes in microbial metabolite profiles during the fermentation process, with lactic and acetic acids, as well as amino acids, amines, and uracil, being the dominant metabolites quantified. Sauerkraut brine did not affect trans-epithelial electrical resistance through a Caco-2 cell monolayer as a measure of gut barrier function. However, significant modulation of inflammatory response after LPS stimulation was observed in PBMCs-Caco-2 co-culture. Sauerkraut brine supported a robust inflammatory response to endotoxin, by increasing TNF-α and IL-6 production while also stimulating the anti-inflammatory IL-10, therefore suggesting positive resolution of inflammation after 24 h and supporting the potential of sauerkraut brine to regulate intestinal immune function.

## Introduction

Fermented foods and beverages, estimated to account for 1/3 of human foods, have been used for millennia as a means of food preservation and to improve the digestibility and nutritional content of foods (Marco et al., 2017; Mota de Carvalho et al., 2018). Sauerkrauts are the most important European fermented vegetable, deriving from the malolactic fermentation of fresh white cabbage (*Brassica oleracea* L. var. *capitata*) salted with 2–3% (w/w) sodium chloride (Di Cagno et al., 2016; Bell et al., 2018). Large-scale industrial production of sauerkraut is supported by the use of bacterial starter cultures; however, homemade and small-scale artisanal products are traditionally obtained by spontaneous fermentation carried out by lactic acid bacteria (LAB) naturally present on the fresh cabbage or in the food processing environment, such as *Weissella* spp., *Leuconostoc mesenteroides, Levilactobacillus brevis, Lactiplantibacillus plantarum*, and *Pediococcus pentosaceus* (Di Cagno et al., 2016; Zabat et al., 2018; Yang et al., 2020).

During food fermentation, bacterial metabolism converts fermentable substrates, mainly carbohydrates and proteins, into biologically active metabolites, including short-chain fatty acids (SCFAs) and biogenic amines. SCFAs mediate several beneficial activities in the gastrointestinal tract, including influencing intestinal motility, acting as an energy source, strengthening the integrity of the intestinal barrier, and anti-oxidative and anti-inflammatory effects (Havenaar, 2011; Fernández-Reina et al., 2018). Biogenic amines and natural polyamines, such as putrescine and putrescine-derived spermine and spermidine, are often present in fermented foods as a result of bacterial decarboxylation of free amino acids and play a role in cell proliferation and differentiation. Natural polyamines influence apoptosis, rate of proliferation, and cellular differentiation, key processes governing the health of the intestinal mucosa (Wunderlichová et al., 2014).

Although human clinical trials are still scarce, consumption of fermented foods has been linked to improved immune and metabolic function, protection against inflammation, and decreased fasting glycemia in type 2 diabetes mellitus (Higashikawa et al., 2010; Moroti et al., 2012). The underlying mechanisms of effects are likely to be due to a combination of bioactive activities associated with the starting food material and an additional contribution from fermentation metabolites. Ingestion of certain probiotic bacteria, including those present in fermented foods, has been shown to induce significant positive improvements in gut barrier function (Lamprecht et al., 2012; Bell et al., 2018; Hiippala et al., 2018). The gut wall forms a tight barrier that prevents bacteria, microbial cell wall components (e.g., lipopolysaccharide or endotoxin), and food antigens from translocating across the intestinal mucosa and triggering inflammation (Ghosh et al., 2020). Increased permeability of the intestinal mucosa (often referred to as “leaky gut”) leads to chronic systemic low-grade inflammation (Cani et al., 2007; Ghosh et al., 2020). This condition, which has been described by Cani et al. (2007) as “metabolic endotoxemia”, has frequently been reported in patients with inflammatory bowel disease (IBD) (Cani et al., 2007; Petit et al., 2012), irritable bowel syndrome (IBS) (Liebregts et al., 2007), diabetes, obesity, and other chronic diseases (Creely et al., 2007). Diet is one of the major factors influencing gut barrier integrity, and several studies investigated the role of food microbial metabolites in regulating intestinal permeability. Some evidence suggests that lactic acid and other organic acids present in fermented products might positively affect gut barrier integrity, thus lowering inflammation.

In this study, an in-depth and multi-disciplinary analysis was conducted to first characterize the fermentation of locally produced organic artisanal sauerkraut and to measure the potential of sauerkraut brine to improve gut health. Specifically, both culture-dependent and -independent microbiological methodologies were used to characterize sauerkraut fermentation, and untargeted proton nuclear magnetic resonance (^1^H-NMR)-based metabolomics was employed for metabolite profiling Additionally, an *in vitro* cell culture model was applied to examine the effect of the sauerkraut brine on gut permeability, as measured by trans-epithelial electrical resistance (TEER) of human colon adenocarcinoma epithelial cell (Caco-2) monolayers and immune function upon endotoxin stimulation of Caco-2 monolayer, peripheral blood immune cells, and co-culture.

## Materials and methods

### Sauerkraut fermentation and sauerkraut brine sampling

Five replicate fermentations were characterized by two artisanal organic producers (SK1 and SK2) in Valle San Felice and Patone, two small villages in the geographical region of Val di Gresta, Trentino (Northern East Italy), between October and November 2019. The sauerkraut fermentation was performed using fresh white cabbage (*Brassica oleracea* L. var. *capitata*) chopped, shredded (ca 0.5 mm wide and 15 cm long pieces), and layered in five separated 500 kg tanks with ~3% sodium chloride. The top of each tank was covered with a nylon coating and a layer of water over the nylon coating was used to keep pressure on the head of the fermenter and maintain anaerobic conditions. Fermentation brine samples were collected from the lower part of the tank using a tap every 24 h for the first 3 days of fermentation and then on days 7, 14, 21, 28, and 35. Samples were taken from five different tanks at each production site using an aseptic technique and stored at −80°C until further analysis. The temperature was measured daily every 15 min using a probe inside each tank and an external probe to record the surrounding ambient temperature. The pH value was measured for each fermentation brine sample using a laboratory potentiometer (BTPH; Bruker, Bremen, Germany).

### CFU/mL of lactic acid bacteria and collection of bacterial isolates

Sauerkraut brine samples were serial decimal diluted in sterile peptone water and spread plated onto de Man, Rogosa and Sharpe (MRS) agar (Oxoid,Thermo Fisher Scientific, Basingstoke, UK), for the calculation of CFU/mL of lactic acid bacteria and isolation of putative lactobacilli. Bacteria were incubated both under anaerobic (using jars with AnaeroGen™ anaerobic system, Thermo Fisher Scientific, Waltham, MA, USA) and aerobic conditions at 30°C for 48 h (Wehr and Frank, 2004). Viable cell counts (CFU/mL) were determined by colony formation on MRS agar using the standard spread plate method. For each different colony morphology, one to three colonies were randomly picked up from countable MRS agar plates for bacterial isolation and purified by subsequent culturing and Gram staining. Each purified isolate was subsequently cultured in MRS and stored at −80°C in 40% glycerol stocks.

### DNA extraction and genotypic identification of sauerkraut brine bacteria

One milliliter of bacterial culture from putative LAB isolates grown overnight in MRS broth was centrifuged at 13,000×*g* for 3 min, the supernatant was discarded, and the pellet was used to prepare bacterial DNA using Instagene Matrix (Bio-Rad, Hercules, CA, USA) extraction kit, following the manufacturer's instruction. RAPD-PCRs were carried out using the primer M13 (5′-GAGGGTGGCGGTTCT-3′) (Gatti et al., 2008). PCR amplification of each sample was carried out in 25 μL of reaction volume, with 2.5 μL of Buffer 10X, 1.5 μL of MgCl_2_ 50 mM, 2 μL of dNTPs 10 mM, 0.1 μL of primer M13 100 μM, 0.2 μL of Taq (5 U/mL), 13.7 μL of ddH_2_O, and 2.5 ng/μL of total DNA. All PCR reagents were purchased from Invitrogen (Thermo Fisher Scientific, Waltham, MA, USA). PCR reactions were carried out using a Verity™ 96-well Thermal Cycler (Thermo Fisher Scientific, Waltham, MA, USA), according to the following protocol: 2 min at 94°C, 40 cycles of 1 min at 94°C, 30 s at 42°C, and 2 min at 72°C, followed by a final extension of 10 min at 72°C (Guzzon et al., 2014). PCR products were separated by electrophoresis on 2% (w/v) agarose gel (Gibco BRL, Cergy Pontoise, France) and stained with ethidium bromide (0.5 μg/L). DNA band patterns were analyzed through the unweighted pair group method arithmetic averages (UPGMA) using the GelCompar II-BioNumerics^®^ software (package version 6.0; Applied Maths, Sint-Martens-Latem, Belgium). Calculation of similarity of the PCR fingerprinting profiles was based on the Pearson product–moment correlation coefficient. Isolates with a similarity coefficient higher than 90% were considered to belong to the same biotype, as described by Gatti et al. (2008). Genotypic identification of different LAB biotypes was carried out by Sanger sequencing of partial 16S rRNA gene. The 16S rRNA gene sequence analysis was performed using P0 (5′-GAGAGTTTGATCCTGGCTCAG-3^··^) and P6 (5′-GGTTACCTTGTTGTTACGA-3^··^) primers, as previously described (Guzzon et al., 2014). Each obtained PCR product was purified with an Exo-SAP-IT™ kit (Thermo Fisher Scientific, Waltham, MA, USA) and sequenced in an ABI PRISM 3100 sequencer (Applied Biosystems™, Thermo Fisher Scientific, Waltham, MA, USA), using the BigDye Terminator v1.1 cycle sequencing kit (Applied Biosystems). Forward and reverse sequence assembly, primer removal, and an ambiguous base correction were performed using DNA baser v5.15 software. For species assignment, sequences were compared using the BLAST algorithm made available by the National Center for Biotechnology Information (NCBI, USA) (Altschul et al., 1990).

### 16S rRNA metataxonomic analysis of sauerkraut brine microbiota

Total DNA extraction from sauerkraut brine was performed using the DNA Blood and Tissue Kit (Qiagen, Hilden, Germany) according to the manufacturer's recommendations and following the protocol “Pretreatment for Gram-Positive Bacteria”. DNA quality was assessed by gel electrophoresis and UV/Vis spectrophotometry. PCR amplification was performed by targeting 16S rRNA gene V3-V4 variable regions with the bacterial primer set 341F (5′-CCTACGGGNGGCWGCAG-3′) and 806R (5′-GACTACNVGGGTWTCTAATCC-3′), as previously reported (Bona et al., 2020). PCR amplification of each sample was carried out in 25 μL of reaction volume, with 12.5 μL of 2X KAPA Hifi HotStart Ready Mix (Kapa Biosystems Ltd., London, UK), 1 μM of each primer, 2 μL of DNA (10 ng/μL), and 9.5 μL of ddH_2_0. All PCR reactions were carried out using a Verity™ 96-well Thermal Cycler, according to the following protocol: 95°C for 5 min and 25 cycles of 95°C for 30 s, 55°C for 30 s, 72°C for 40 s, with a final elongation step of 72°C for 5 min. PCR products were checked by gel electrophoresis and cleaned using an Agencourt AMPure XP system (Beckman Coulter, Brea, CA, USA), following the manufacturer's instructions. After seven PCR cycles (16S metataxonomic Sequencing Library Preparation, Illumina), Illumina adaptors were attached (Illumina Nextera XT Index Primer). Libraries were purified using Agencourt AMPure XP (Beckman Coulter, Brea, CA, USA), and then sequenced on an Illumina^®^ MiSeq (PE300) platform (MiSeq Control Software 2.0.5 and Real-Time Analysis software 1.16.18, Illumina, San Diego, CA, USA). All analyses were performed in R (version 4.0.2). Sequences obtained from Illumina sequencing were imported, filtered, denoised, merged, and chimeras removed using the DADA2 package (version 1.16; Callahan et al., 2016). Taxonomy was assigned to amplicon sequence variants (ASVs) using the Silva reference database (version 138.1). Multiple sequence alignment (MSA) was created using the DECIPHER package (v2.16.1; Wright, 2015), and the phylogenetic tree was inferred using the packages APE (version 5.4; Studier and Keppler, 1988) and PHANGORN (version 2.5.5; Schliep, 2011). The phylogenetic tree, read count data, assigned ASVs, and sample metadata were imported into the PHYLOSEQ package (v1.32; McMurdie and Holmes, 2013) for downstream analysis. Changes in α-diversity between sauerkraut fermentation time points were compared using Kruskal–Wallis test. The principal component analysis (PCoA) with multidimensional scaling (MDS) was performed on log-transformed data using Bray–Curtis dissimilarity.

### ^1^H-NMR analysis of sauerkraut brine metabolites

From each sauerkraut brine sample, 900 μL were mixed with 100 μL of deuterium oxide (99.9 %) containing 0.03% 3-(trimethylsilyl)propionic-2,2,3,3-d4 acid sodium salt or TMSP-d4 (Deutero GmbH, Kastellaun, Germany) and vortexed for 15 s, filtered using sterile Sartorius 0.22 μm PVDF syringe filters (Thermo Fisher Scientific, Waltham, MA, USA), and transferred to the 5 mm NMR tube. ^1^H-NMR spectra were recorded on Bruker Avance Neo 600 (base frequency 600 MHz for ^1^H nuclei) spectrometer, equipped with a broadband Z-gradient probe (5 mm sample tubes) and SampleCase 24-position autosampler (Bruker BioSpin GmbH, Rheinstetten, Germany). The spectra were acquired and processed using Topspin 4.1.1 software in the automation mode with Icon NMR 5.2.1. The deuterium lock signal was optimized for the 9:1 mixture of H_2_O and D_2_O (v/v). All proton NMR spectra were recorded using the noesygppr1d pulse sequence with automatic adjustment of water signal suppression frequency (o1p), and the power level utilized for pulse was 47.10 dB (25 Hz suppression window). The size of the spectrum (sweep width, SW) was 20.8 ppm, the time domain (TD) consisted of 65,536 (64K) data points, the number of scans (NS) was 64 and the number of dummy scans (DS) was 4, the time for relaxation delay (D1) was 10 s, receiver gain (RG) for all spectra was fixed at 101, and baseopt digitization mode was used. Acquisition of each spectrum was preceded by automatic adjustment of the probe (ATMA routine) and automatic shimming (TOPSHIM). Spectra were processed in the TopSpin software with the size of the real spectrum (SI) set to 131,072 (128K, 2xTD) data points and apk0.noe phase correction au program was applied automatically to each spectrum.

Quantitative analysis was performed using Assure-NMR software with the external standard technique (ERETIC or Electronic REference To access *In vivo* Concentrations; Hong et al., 2013), with the 2 mM sucrose reference solution in a 9:1 mixture of H_2_O and D_2_O (v/v) used as the external standard.

Identification of metabolites was performed either manually based on literature data (Tomita et al., 2017) or in automation mode in AssureNMR utilizing the Human Metabolome Database (HMDB; Wishart et al., 2018) and the BBIOREFCODE database of NMR metabolites.

### Correlation analysis between bacteria and metabolites

Sparse canonical correlation analysis (sparse CCA) was performed to quantify what features (bacteria and metabolites) are associated with fermentation time and producers (Witten et al., 2009). Therefore, measurements were preprocessed and normalized to weaken heavy tails, and then sparse CCA of the PMA package (version 1.2.1) was applied to capture the most covariant features (Witten and Tibshirani, 2009; Witten et al., 2009). Recovered features were used as input for a principal component analysis (PCA) using the dudi.pca function of the ade4 package (version 1.7-16; Dray and Dufour, 2007) and visualized using the factorextra package (version 1.0.7; Kassambara and Mundt, 2020).

### *In vitro* model of the intestinal epithelium

#### Peripheral blood mononuclear cell (PBMC) isolation and maintenance

Peripheral blood mononuclear cells (PBMCs) were isolated the day before the experiment from buffy coat blood samples from healthy donors (*n* = 3), donated by the Transfusion Unit of Santa Chiara Hospital, Trento, Italy, by Lymphoprep™ density gradient centrifugation (Thermo Fisher Scientific, Waltham, MA, USA). The experimental plan was approved by the local Ethical Committee of Azienda Provinciale dei Servizi Sanitari (APSS, Santa Chiara Hospital, Italy; approval document n. 401/2015). The study was designed in conformity with the international recommendation (Dir. EU 2001/20/EC) and its Italian counterpart (DM 15 Luglio 1997; D.Lvo 211/2003; D.L.vo 200/2007) for clinical trials and following the Declaration of Helsinki, to assure the protection and care of subjects involved. Briefly, fresh human blood was diluted 1:1 in PBS 1X and then gently transferred in sterile 50 mL tubes (Sarstedt, Nümbrecht, Germany) containing Lymphoprep™ in 2:1 proportion (Thermo Fisher Scientific, Waltham, MA, USA). Samples were centrifuged at 400 x *g* without break at room temperature for 20 min. PBMCs were collected from the ring surrounding the Lymphoprep™ layer, and then washed twice in unsupplemented RPMI 1640 (Merck Life Science S.r.l., Milano, Italia) and once in PBS 1X (Merck Life Science). PBMCs were resuspended in RPMI 1640 supplemented with 10% decomplemented (56°C, 60 min) fetal bovine serum (Lonza, Basel, Switzerland), 1% penicillin (100 U)-streptomycin (100 μg)/mL, 1% 200 mM L-glutamine, and 1% 100 mM sodium pyruvate (Biological Industries, Beit-Haemek, Israel), described below as “complete RPMI”, and seeded at a concentration of 2 × 10^6^ cells/cm^2^ in the basolateral compartment of the co-culture inserts. Cells were maintained in a humidified atmosphere of 5% CO_2_ in air at 37°C for 18 h before using them in the Caco-2 co-culture experiment.

#### Caco-2 cell cultivation and maintenance

Human epithelial colorectal adenocarcinoma Caco-2 cell line (ATCC^®^ HTB-37™, number of passages between 50 and 60) was grown in Dulbecco's Modified Eagle's Medium (DMEM; Biological Industries) with high glucose (4.5 g/L) (Lonza) supplemented with 20% decomplemented (56°C, 60 min) fetal bovine serum (Lonza), 1% penicillin (100 U)-streptomycin (100 μg)/mL (Biological Industries), 1% 10 mM non-essential amino acids (Euroclone, Milan, Italy), 1% 200 mM of L-glutamine, and 0.1% amphotericin 0.25 μg/mL (Biological Industries) described below as “complete DMEM”. Cells were maintained in T-75 cm^2^ flasks (Sarstedt) in a humidified atmosphere of 5% CO_2_ in air at 37°C and passaged when they reached 70% confluence (estimated by viewing the cells under the microscope) using 0.05% trypsin−0.5 mM EDTA (Lonza). The medium was refreshed every second day.

#### Trans-epithelial electrical resistance (TEER)

Trans-epithelial electrical resistance (TEER) of Caco-2 monolayer before and after 24 h of incubation with 10% sauerkraut brine was assessed as a measure of the integrity of the gut epithelial barrier. The apical compartment of transwell inserts (0.4 μm pore size; Falcon, Sacco s.r.l, Cadorago, CO, Italy) was coated with a 200 μg/mL rat tail collagen Type I (Sigma Aldrich, Merck Life Science) solution and left to dry under sterile laminar flow. Inserts were put into six-well plates (Falcon, Sacco s.r.l, Cadorago, CO, Italy). Caco-2 cells were harvested from T-75 cm^2^ flasks (Sarstedt), using 0.05% trypsin−0.5 mM EDTA (Lonza) and resuspended in complete DMEM to obtain a cell suspension of 1 × 10^5^ cells/mL. About 2.5 mL of Caco-2 cell suspension was added to transwell inserts (apical compartment), and 1.5 mL of complete DMEM was added to the basolateral chamber. Caco-2 cells were grown for 13 days until a tight monolayer was formed (i.e., when TEER measure was stable for 2 consecutive days, after measuring it daily from day 9). On the day of the experiment, DMEM was removed from both the apical and the basolateral compartment of the transwells. Pooled sauerkraut brine samples from both producers (SK1 and SK2) collected at 35 days of fermentation were thawed on ice, filter-sterilized using Sartorius 0.22 μm filters (Thermo Fisher Scientific, Waltham, MA, USA), and added to complete DMEM at 10% v/v. The solutions of 10 mM propionic acid and 7% ethanol were prepared in complete DMEM on the day of the assay, and they were used as positive and negative controls, respectively. The experimental layout is schematically illustrated in Supplementary Figure S1.

The TEER was measured using an epithelial volt-ohm-meter (EVOM, World Precision Instruments Inc., Sarasota, FL, USA). Six-well plates containing the transwell inserts were left at room temperature for exactly 25 min prior to TEER measurements. The integrity of cell monolayers was assessed just before the addition of testing substrates by measuring TEER (resistance_0h_), as described below. The media were then removed from the basolateral and apical compartments of the transwells, and the control or test treatments were added to the apical compartment. TEER was measured after 24 h of incubation (resistance_24h_). The TEER was calculated using the following equation, as described in previous works (Coates et al., 2007; Anderson et al., 2010; Srinivasan et al., 2015):


$$\begin{array}{l} \text{TEER}\left(\Omega {\text{cm}}^{2}\right)\;\text{= resistance }\left(\Omega \right)\;\times \text{membrane area}\left(\text{c}{\text{m}}^{2}\right). \end{array}$$

Here, the area of the semipermeable membrane was 9.6 cm^2^. The change in TEER for each insert was then calculated using the following formula:


$$\begin{array}{l} {\text{Change in TEER }\left(\text{\%}\right)=\text{TEER}}_{24\text{h}}\left(\Omega \;cm^{2}\right)/{\text{TEER}}_{0\text{h}}\left(\Omega \;cm^{2}\right)\\ \;\times \;100\%, \end{array}$$

where TEER_24h_ and TEER_0h_ represent TEER after 24 h of treatment and TEER at baseline, respectively. RNA and total protein lysate were collected from Caco-2 cells at 0, 3, and 6 h. This experiment was repeated three times.

### Anti-inflammatory effect assay

To evaluate the anti-inflammatory effects of sauerkraut brine, an *in vitro* assay using a Caco-2/PBMCs co-culture model was performed (Supplementary Figure S1), according to previous studies (Bianchi et al., 2019). Caco-2 cells were harvested from T-75 cm^2^ flasks (Sarstedt) using 0.05% trypsin−0.5 mM EDTA (Lonza) and resuspended in complete DMEM to obtain a cell suspension of 1 × 10^5^ cells/mL. About 2.5 mL of Caco-2 cell suspension was added to transwell inserts (apical compartment) placed on 35-mm diameter six-well plates (Falcon, Sacco s.r.l, Cadorago, CO, Italy), and 1.5 mL of complete DMEM was added to the basolateral compartment. Caco-2 cells were grown for 13 days until a tight monolayer was formed (i.e., when TEER measure was stable for 2 consecutive days, after measuring it daily from day 9). PBMCs/mL were isolated and maintained, as described above, and then seeded to 35-mm diameter six-well plates (Falcon) at a concentration of 2 × 10^6^ cells/cm^2^. Complete adherence of PBMCs to the well was ensured by overnight incubation at 37°C at 5% CO_2_. On the day of the experiment, transwell inserts containing Caco-2 were added to the wells containing PBMCs. Basolateral RPMI medium was refreshed, and DMEM medium was replaced with complete RPMI in the presence or absence of 10% sauerkraut brine (pooled SK1 and SK2 samples collected at 35 days of fermentation, thawed on ice, and filter-sterilized using Sartorius 0.22 μm filters; Thermo Fisher Scientific, USA). Similarly, 10 mM propionic acid and 7% ethanol were prepared in complete RPMI on the day of the assay, and they were used as positive and negative controls, respectively. This co-culture system was incubated for 2 h at 37°C at 5% CO_2_ (baseline). The co-culture system was then challenged with 10 ng/mL of lipopolysaccharide (LPS) in ddH_2_O added to the basolateral compartment and incubated for 24 h at 37°C at 5% CO_2_. Supernatants were collected for cytokine measurements at baseline and after 24 h of incubation with sauerkraut brine or control medium (Ctrl) added at 10% v/v. Trizol lysate for future RNA extraction was collected from PBMCs and Caco-2 cells after 0, 3, and 6 h of incubation with sauerkraut brine or Ctrl. This experiment was repeated three times with three different PBMC donors.

### Cytokine quantification

Upper and basolateral supernatants were collected at 0 and 24 h after the LPS challenge, following 2 h of pre-incubation in the presence or absence of 10% sauerkraut brine. Supernatants were centrifuged for 5 min at 18,000 xg to pellet the cell debris and stored at −80°C. The release of IL-1β, IL-6, IL-10, and TNF-α into the apical and basolateral compartments was quantified in the supernatants using a cytokine magnetic bead-based panel (Milliplex MAP kit, Millipore Corp., Billerica, MA, USA) and measured by a Magpix^®^ instrument (Luminex, Texas, USA) and xPONENT^®^ software (version 4.2, Luminex Corp, Austin, Texas, US) according to the manufacturer's instructions. Blanks and standard curves were included on each plate (Merck, Millipore, 2022).

### Gene expression analysis

For the extraction of RNA from collected PBMCs and Caco-2, the culture medium was first removed and 500 μL of TRIzol reagent was added (Thermo Fisher Scientific, Waltham, MA, USA). Samples were stored at −80°C. Total RNA was isolated from cultured cells according to the manufacturer's recommendations (Thermo Fisher Scientific, Waltham, MA, USA). The extracted total RNA was quantified using a Nanodrop 8,000 Spectrophotometer (Thermo Fisher Scientific, Waltham, MA, USA), and RNA quality was assessed using a 2,200 TapeStation (Agilent Technologies, Santa Clara, CA, USA). The mRNA samples of high quality (RNA Integrity Number, RIN > 8) were used for retrotranscription. Reverse transcription was performed with a High-Capacity cDNA Reverse Transcription Kit (Applied Biosystems^TM^, Thermo Fisher Scientific, USA) in a 20 μL reaction volume containing 10 μL of RNA template (5 ng/μL), 2.0 μL of 10X RT Buffer, 0.8 μL of 25X dNTP Mix (100 mM), 2.0 μL of 10X RT random primers, 1.0 μL of MultiScribe™ Reverse Transcriptase, and 4.2 μL of DEPC-treated water. After transcription, cDNA was stored at −20°C until quantitative real-time PCR (RT-PCR) was performed. The expression level of inflammatory genes was determined by RT-PCR using TaqMan^®^ Gene Expression Assays (Applied Biosystems™) and a ViiA™ 7 System (Thermo Fisher Scientific, MA, USA). RT-PCR was carried out in 20 μL of volume reactions prepared following the manufacturer's instructions and containing 10 μL of KAPA PROBE FAST qPCR Master Mix 2x Universal (Kapa Biosystems), 0.4 μL of 50x Rox Low (10 ng/μL), 1 μL of TaqMan Assay, 2 μL of cDNA (10 ng/μL), and 6.6 μL of H_2_O. Reactions were carried out in triplicate under the following conditions: 95°C for 1 min, followed by 40 cycles at 95°C for 1 s and 60°C for 20 s. Ct values for each sample were normalized against the geometric mean Ct values obtained for two housekeeping genes, 18S and GAPDH. Gene expression was therefore expressed as the relative fold change 2–ΔΔCt, where ΔCt was obtained by subtracting the geometric mean Ct for the two reference housekeeping genes 18S and GAPDH from the Ct of the tested gene, and ΔΔCt represented the difference between ΔCt of cells incubated with sauerkraut water compared to the ΔCt of control samples (medium with no treatment, Ctrl).

### Quantification of tight junction proteins by Western blot

The analyses were performed on the Caco-2 cells from the co-culture system, following the method described by Bianchi et al. (2019) with some modifications. Briefly, the monolayers were rinsed two times with ice-cold PBS and then covered with 350 μL of lysis buffer (20 mM Tris-HCl, pH 7.5, 150 mM NaCl, 1 mM EDTA, 1 mM EGTA, 1% Triton, 2.5 mM sodium pyrophosphate, 1 mM β-glycerophosphate, 1 mM Na3VO4, 1 mM NaF, and 2 mM imidazole) supplemented with a protease inhibitor cocktail (Complete, Mini, EDTA-free, Roche, Monza, Italy). Total cell lysates were collected in 2.0 mL tubes, sonicated, and centrifuged at 14,000×*g* for 10 min at 4°C to eliminate cell debris. Protein concentration was determined by performing a Bicinchoninic Acid (BCA) protein assay using Pierce™ BCA Protein Assay Kit (Thermo Fisher Scientific, USA). Proteins were precipitated by the addition of four volumes of cold acetone (Sigma-Aldrich, Merck Life Science) followed by overnight incubation at 4°C. Samples were pelleted and resuspended in loading buffer (63 mM Tris-HCl, pH 6.8, 2% SDS, 10% glycerol, 0.1% 2-mercapto-ethanol, and 0.005% bromophenol blue) to a final concentration of 35 μg protein/15 μL. This mixture was boiled at 85°C for 15 min for soluble protein or at 70°C for 5–10 min for multi-pass membrane proteins. Samples were loaded on 12% SDS-polyacrylamide gel (35 μg proteins/well), and proteins were separated for 1.30 h at 100 V. Proteins were blotted on PVDF membrane (Immobilon-P, Millipore, Millipore Merck Corporation, Burlington, MA, USA) for 1 h at 100 V and 4°C. After transfer, PVDF membrane was rinsed using TRIS-buffered saline (TBS) and then incubated in TBS with 0.05%Tween (TBS-T) and 5% skim milk powder (Oxoid) solution for 1 h at room temperature (RT). The membrane was washed three times in TBS-T and then exposed overnight at 4°C to primary monoclonal antibodies (Santa Cruz Biotechnology, Dallas, TX, USA; initial concentration 200 μg/mL) diluted 1:1,000 in TBS-T with a 1% skim milk powder solution. After three washes of 10 min each in TBS-T, membranes were exposed to the HRP-conjugated secondary antibodies in TBS-T with 0.5% skim milk powder solution for 1 h at RT. After three washes of 10 min each in TBS-T and one wash in PBS-T, visualization of protein bands was performed by combining 10X CN/DAB concentrate with the stable peroxide substrate (1:10 proportion). The development reaction was stopped by rinsing the membrane with H_2_O. Quantification of band intensity was done by employing the ImageJ Software (designed at the National Institutes of Health, https://imagej.nih.gov/ij), as previously described by Gallo-Oller et al. (2018). Each band was individually selected and circumscribed with the region of interest (ROI) selection under the “Gels” function, followed by quantification of the acquired data. Pixel density was inverted for all data and background noise was removed, following the manufacturer's instructions (https://imagej.nih.gov/ij/docs/guide/user-guide.pdf). Data were expressed as a normalized ratio (fold-changes) to β-actin and to control samples (cells incubated with complete DMEM medium as control).

### Statistical analysis

All statistical analysis was performed using R studio version 3.6.2. The normal distribution of data was assessed by Shapiro–Wilk's test. Differences in microbial metabolites over fermentation time within the same producer were analyzed by Kruskal–Wallis test, followed by the *post-hoc* Dunn's test with Benjamini–Hochberg false discovery rate (FDR) *p*-value correction. 16S rRNA metataxonomic data were analyzed as detailed in the Materials and Methods section. Statistical significance between TEER measures, Magpix^®^ cytokine quantification, gene expression, and Western blot data was performed by one-way ANOVA. After FDR correction, a *p*-value < 0.05 was considered to be statistically significant. All data are expressed as the mean ± standard deviation (SD).

## Results

### Characterization of sauerkraut fermentation

**Figure 1:**
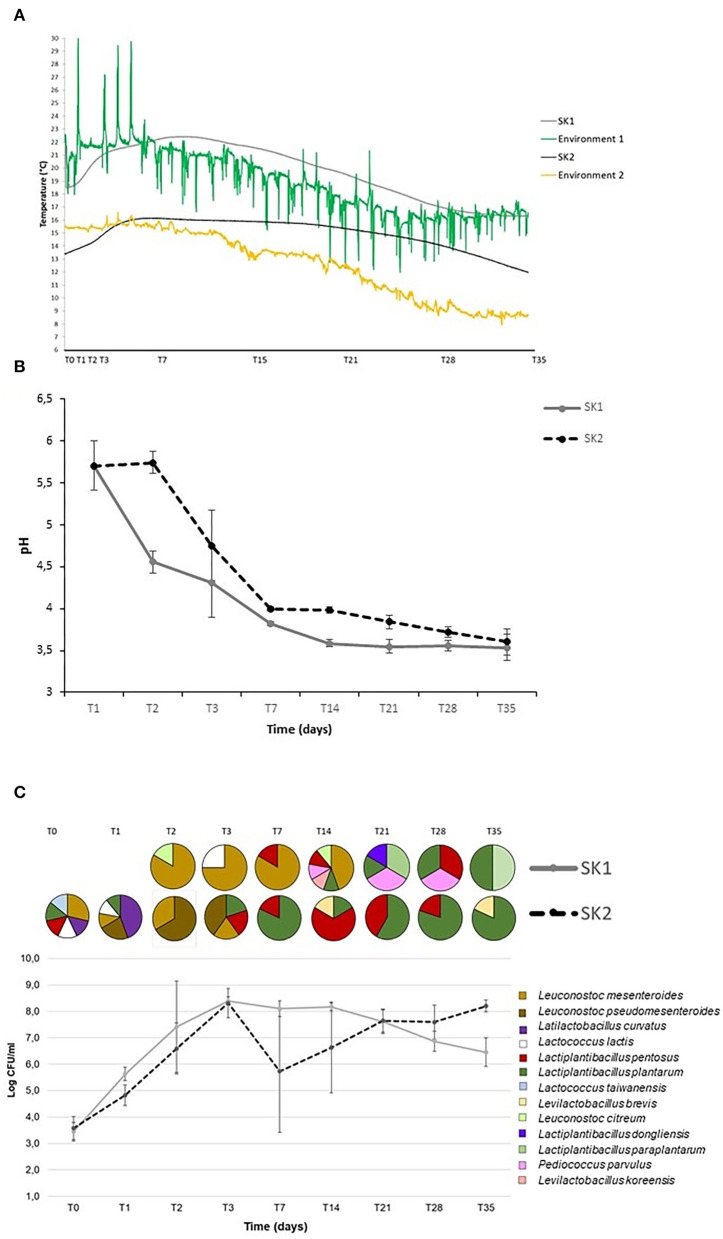
Temperature (°C) **(A)**, pH **(B)**, and bacterial counts (CFU/mL) **(C)** during sauerkraut fermentations (mean and standard deviation *n* = 5 tanks for SK1 and SK2). The circles represent the percentage of bacterial strains isolated and identified at each time point.

Changes in temperature, pH, and CFU/mL of LAB are reported in Figure 1. Temperature (°C) inside all tanks changed significantly over the fermentation process (T0: 19.1 ± 1.2°C; T3: 21.5 ± 0.3°C; T7: 22.1 ± 0.3°C; T14: 20.7 ± 0.5°C; T21: 18.7 ± 0.6°C; T28: 16.5 ± 0.2°C; T35: 16.3 ± 0.01 for SK1, and T0: 13.5 ± 0.1°C; T3: 15.7 ± 0.5°C; T7: 16.0 ± 0.1°C; T14: 15.7 ± 0.1°C; T21: 15.0 ± 0.3°C; T28: 13.0 ± 0.6°C; T35: 9.1 ± 0.1°C for SK2, Figure 1A). As expected, the temperature increased rapidly during the first week of fermentation and gradually decreased after day 18 for both producers. Notably, the temperature recorded in the room where tanks were stored underwent many more changes, both daily and weekly, without affecting the temperature inside the fermentation tanks.

The pH decrease was rapid during the first 7 days of fermentation in all tanks, dropping from 5.7 ± 0.2 at T1 to 3.8 ± 0.1 at T7 in SK1 (*p* = 0.0001) and from pH 5.7 ± 0.3 at T1 to pH 4.0 ± 0.02 at T7 in SK2 (*p* = 0.0002; Figure 1B). Thereafter, pH declined at a slower rate and remained unchanged after day 21. Similar pH profiles were obtained for samples from all five tanks for both producers.

For both producers, the Log CFU/mL of LAB increased rapidly after the first days of fermentation and reached high density by day 3 (T0: 3.4 ± 0.4, T1: 5.6 ± 0.3, T2: 7.4 ± 1.7, T3: 8.4 ± 0.2, Log CFU/mL, T0 vs. T1: *p* = 0.0004, T1 vs. T2: *p* = 0.012, T2 vs. T3: *p* = 0.00001 for SK1; T0: 3.6 ± 0.4, T1: 4.8 ± 0.4, T2: 6.6 ± 1.0, T3: 8.3 ± 0.6, Log CFU/mL, T0 vs. T1: *p* = 0.0003; T1 vs. T2: *p* = 0.0006; T2 vs. T3: *p* = 0.00004 for SK2), when the inside-tank temperature significantly increased from 19.1 ±1.16°C to 21.5 ± 0.3°C for SK1 and from 13.5 ± 0.06 to 15.7 ± 0.5°C for SK2 (*p* < 0.05). On day 35, average LAB counts were 6.4 ± 0.5 Log CFU/mL in SK1 and 8.2 ± 0.2 in SK2 (Figure 1C). The decline in LAB CFU/mL observed after day 3 might be due to the high acidity (Figure 1C). Two of the five tanks sampled in SK2 showed a striking reduction in LAB counts, explaining the larger SD for LAB counts from this producer.

### Identification of bacterial isolates

A total of 251 isolates were obtained from MRS agar plates, and RAPD-PCR fingerprinting was performed to preliminarily categorize bacterial isolates into genetically similar phylotypes. One isolate from each biotype was then further classified by partial 16S rRNA gene sequencing. Of the 251 isolates, 31 did not grow after plate isolation and were discarded. RAPD-PCR with the M13 primer generated 220 fingerprints that were observed by agarose gel electrophoresis. The Bionumerics^®^ analysis clustered the 220 fingerprints into 133 biotypes with a 90% similarity index. In order to characterize the main species driving sauerkraut fermentation at the species level, 64 biotypes from three of the five tanks of producers SK1 and SK2 were selected for sequencing: 31 biotypes related to 43 bacteria isolated from producer SK1 and 33 biotypes related to 57 isolates from producer SK2. Cluster analysis of RAPD-PCR profiles showed that, at a similarity level of 90%, *Lactiplantibacillus plantarum/paraplantarum* and *Leuconostoc mesenteroides* accounted for 72% of all isolates from producer SK2 and *Lactiplantibacillus plantarum/pentosus* for the 67% of all isolates at producer SK1. Some species were distinctive or specific characteristics of a producer. *Leuconostoc curvatus, Levilactobacillus brevis*, and *Lactococcus taiwanensis* were found only in the samples obtained from producer SK1, and *Lactiplantibacillus dongliensis, Leuconostoc citreum, Levilactobacillus koreensis*, and *Pediococcus parvulus* only in samples obtained from producer SK2 (Figure 1C). The culture-based microbiology characterization suggests the existence of three stages in sauerkraut fermentations. The first stage, from T0 to T7, was characterized by a lowering pH (from pH 5.7 ± 0.2 to pH 3.8 ± 0.1 in SK1 and from pH 5.7 ±0.3 to pH 4.0 ± 0.02 in SK2), rising temperature (from 19.1 ± 1.2 to 22.1 ± 0.3°C in SK1 and from 13.5 ± 0.1 to 16.0 to 0.1°C in SK2), rising bacterial counts (mean Log[CFU/mL] ± SD) from 3.4 ± 0.4 on T0 to 8.1 ± 0.3 on T7 for SK1 and from 3.6 ± 0.4 to 5.7 ± 2.3 in SK2, and dominance of *Leuconostoc mesenteroides/pseudomesenteroides* in both SK1 and SK2 samples. The second stage, from T7 to T14, was characterized by a constantly low pH (pH 3.8 ± 0.1 on T7 and pH 3.6 ± 0.04 on T14 in SK1 and pH 4.0 ±0.02 on T7 and pH 4.0 ± 0.04 on T14 in SK2), high bacterial counts [mean Log[CFU/mL] ± SD] (8.1 ± 0.3 on T7 and 8.2 ±0.1 on T14 in SK1; 5.7 ± 2.3 on T7 and 6.6 ± 1.7 on T14 in SK2), lowering the temperature (°C) from 22.1 ± 0.3 to 18.7 ± 0.6 in SK1 and from 16.0 ± 0.1 to 15.0 ± 0.3 in SK2, and unclear bacterial species dominance. Finally, the third stage, from T14 to T35, was characterized by almost constant low pH (pH 3.6 ± 0.04 on T14 and pH 3.5 ± 0.04 on T35 in SK1; pH 4.0 ± 0.04 on T14 and pH 3.6 ± 0.15 on T35 in SK2), slightly lowering the temperature (°C) from 18.7 ±0.6 to 16.3 ± 0.4°C in SK1 and from 15.0 ± 0.3 to 12.0 ± 0.2°C in SK2, nearly constant bacterial counts (mean Log[CFU/mL] ± SD; 8.2 ± 0.1 on T14 and 6.5 ± 0.5 on T35 in SK1; 6.6 ± 1.7 on T14 and 8.2 ± 0.2 on T35 in SK2), and dominance of *Lactiplantibacillus plantarum/paraplantarum* in samples obtained from both producers.

### Microbial ecology by 16S rRNA metataxonomic analysis

**Figure 2:**
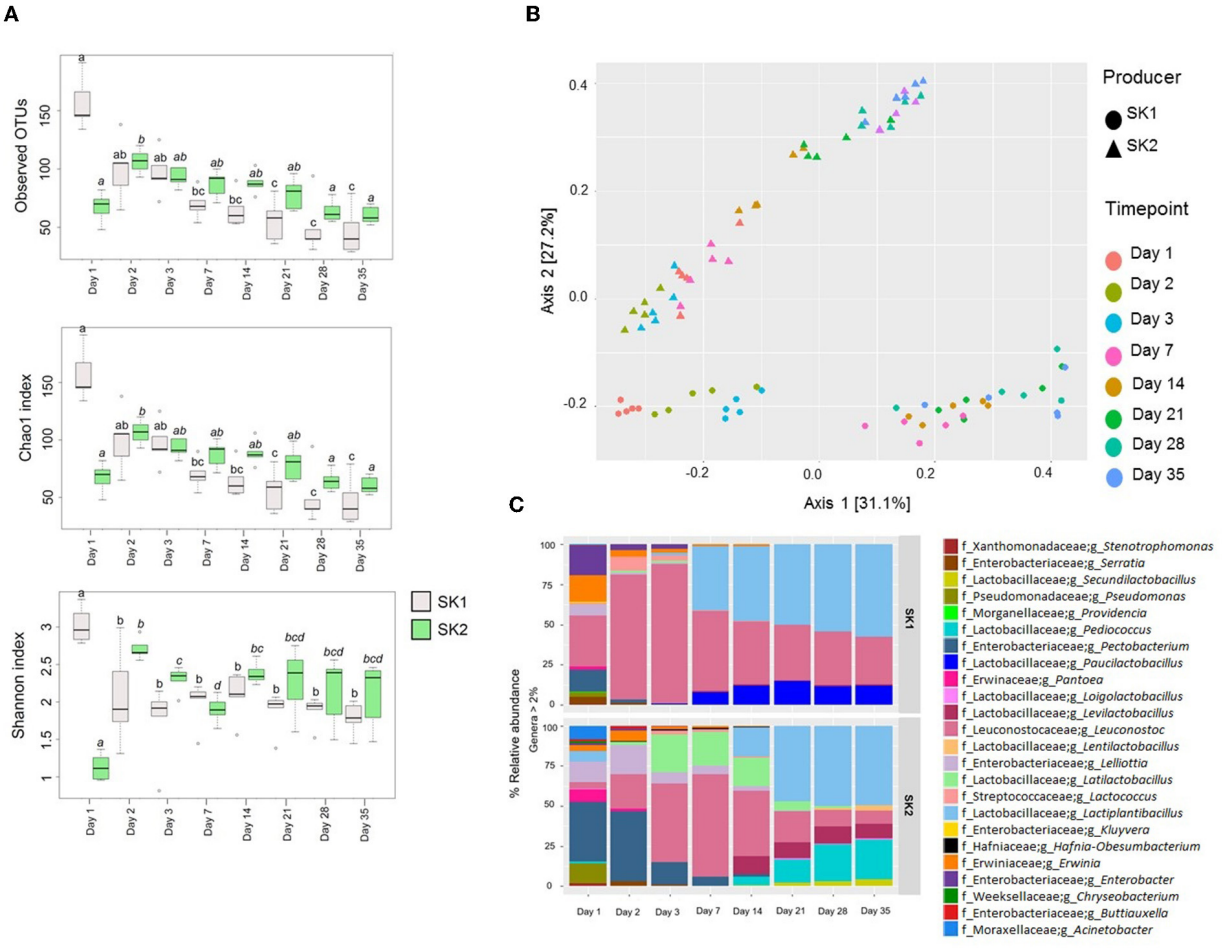
Metataxonomic analysis of sauerkraut fermentation brine. **(A)** Bacterial α-diversity using observed OTUs, Chao1, and Shannon index from day 1 to day 35 of sauerkraut fermentation from both producers (SK1 and SK2). The line inside the box represents the median, and the whiskers from either side of the box represent the first and the third quartiles, respectively. °=outliers. Different superscript letters indicate statistical significance within the same producer. **(B)** Principal component analysis (PCoA) representing the bacterial β-diversity according to Bray–Curtis dissimilarity index. Different colors and shapes indicate different time points and producers, respectively, as described in the legend. **(C)** Mean percentage relative abundance of the most abundant genera (>2%) at days 1, 2, 3, 7, 14, 21, and 35 of fermentation (*n* = 5 tanks).

Community partial 16S rRNA gene sequencing produced an average of 61,599.55 ± 12,467.47 good quality sequences per sample. Observed OTUs, Chao1, and Shannon α-diversity indices are shown in Figure 2A. In SK1, bacterial richness significantly decreased over 35 days of fermentation, according to observed OTUs and Chao1 index (observed OTUs: day 1 vs. day 7, *p* = 0.018; day 1 vs. day 21, *p* = 0.003; day 1 vs. day 35, *p* = 0.001; Chao1: day 1 vs. day 7, *p* = 0.019; day 1 vs. day 21, *p* = 0.003; day 1 vs. day 35, *p* = 0.001). In SK2, bacterial richness significantly increased between days 1 and 2 (observed OTUs: *p* = 0.047; Chao1: *p* = 0.048) and then significantly decreased until day 35 (day 2 vs. day 35; observed OTUs: *p* = 0.0195; Chao1: *p* = 0.0199). Shannon index significantly decreased during the first 3 and 7 days of fermentation, respectively, in SK1 (day 1 vs. day 3, *p* = 0.0079) and SK2 (day 1 vs. day 7, *p* = 0.0079). After day 3, a slight increase in α-diversity was observed for SK1, but this result did not reach statistical significance. Similarly, an increase in Shannon α-diversity index was observed for SK2 on day 14 when compared to day 7 (*p* = 0.0079). This increase in Shannon index was concomitant with a drop in LAB Log[CFU/mL] observed for SK2 after day 7. In order to highlight the differences in bacterial composition following sauerkraut fermentation over time, β-diversity was plotted using Bray–Curtis dissimilarity matrix (Figure 2B). Significant changes in sauerkraut brine microbiota composition were observed, with a significant shift from day 1 to day 35 (*p* < 0.01). The same trend was observed for both producers. The relative abundance (%) of genera in sauerkraut brine is shown in Figure 2C. The number of identified genera dramatically decreased from day 1 to day 2, and then slowly further decreased until day 35 when a lower number of genera was observed for both producers compared to the beginning of fermentation (T1, SK1: 8.8 ± 1.5; SK2: 9.2 ± 1.3; T2, SK1: 4.8 ± 2.6; SK2: 6.6 ± 1.1; T3, SK1: 4.4 ± 2.1; SK2: 6.4 ± 1.1; T7, SK1: 3.8 ± 0.8; SK2: 5.2 ± 0.8; T14, SK1: 3.8 ± 0.8; SK2: 6.6 ± 1.3; T21, SK1: 3.2 ± 0.5; SK2: 5.6 ± 1.3; T28, SK1: 3.2 ± 0.5; SK2: 5.8 ± 1.8; T35, SK1: 3.2 ± 0.5; SK2: 5.6 ± 1.1; mean number of genera in the five tanks ± SD). With reference to the relative abundance of the genera and families of bacteria found throughout the fermentation, it can be mentioned that on day 1, a significantly higher *Enterobacteriaceae* was observed in producer 1 (SK1: 24.44 ± 5.83%) compared to day 2 (SK1: 10.09 ± 5.41%). Higher *Pseudomonadaceae* were observed in both producers on day 1 (SK1: 3.33 ± 0.21 %; SK2: 2.25 ± 2.25%) compared to day 2 (SK1: not detected; SK2: not detected). *Pantoea*, a colonizer of the surface of vegetables and often found on *Brassica* leaves, was recovered from both producers on day 1 (SK1: 3.34 ± 1.62%; SK2: 6.67 ± 1.89%) and also on day 2 in SK2 (2.43 ± 0.13%). On day 35, the mean relative abundance ± SD of the following dominant genera was observed in both SK1 and SK2: *Lactiplantibacillus* (SK1: 56.19 ± 15.21%; SK2: 47.75 ± 12.68%)*, Leuconostoc* (SK1: 29.00 ± 12.61 %; SK2: 8.05 ± 2.85%*), Pediococcus* (SK2: 24.16 ± 8.12%), *Levilactobacillus* (SK2: 8.67 ± 5.86%), *Paucilactobacillus* (SK1: 11.66 ± 5.04%), and *Secundilactobacillus* (SK2: 4.95 ± 2.18%). In SK1, *Lactiplantibacillus, Leuconostoc*, and *Paucilactibacillus* became the dominant genera since day 7 and persisted until day 35, as shown in Figure 1. In SK2, this shift in microbiota composition happened after day 14, when *Lactobacillaceae* (SK1: 86.13 ± 3.82%) became more prevalent than *Enterobacteriaceae* (SK1: 5.52 ± 1.55%). All data are referred to as mean relative abundance ± SD.

### ^1^H-NMR metabolite analysis

**Figure 3:**
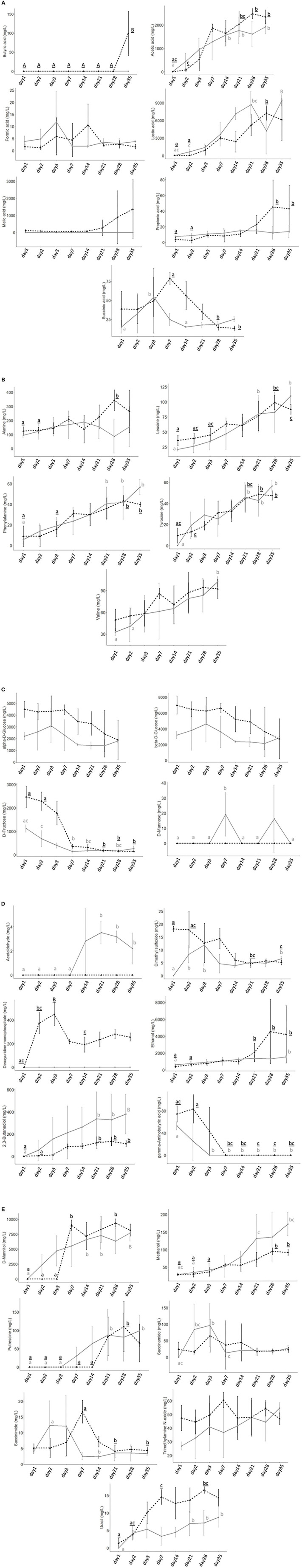
Individual line plots of microbial metabolites identified by ^1^H-NMR analysis, divided into **(A)** Organic acids, **(B)** Amino acids, **(C)** Sugars, **(D)** Other compounds, and **(E)** Other compounds. The plots compare the concentration (mg/L) changes between all experimental time points, after 1, 2, 3, 7, 14, 21, 28, and 35 days of fermentation. Different colors and line shapes indicate different producers: — SK1; - - - SK2. Plots represent the mean metabolite concentration (*n* = 5 tanks for each producer), and the error bars correspond to the standard deviation. Different superscript letters indicate statistical significance among different time points, within the same producer (*p* < 0.05) and capital letters indicate a *p*-value < 0.001. Gray-colored letters refer to SK1, and black underlined letters refer to SK2. No letter indicates no statistical significance after FDR correction.

**Figure 4:**
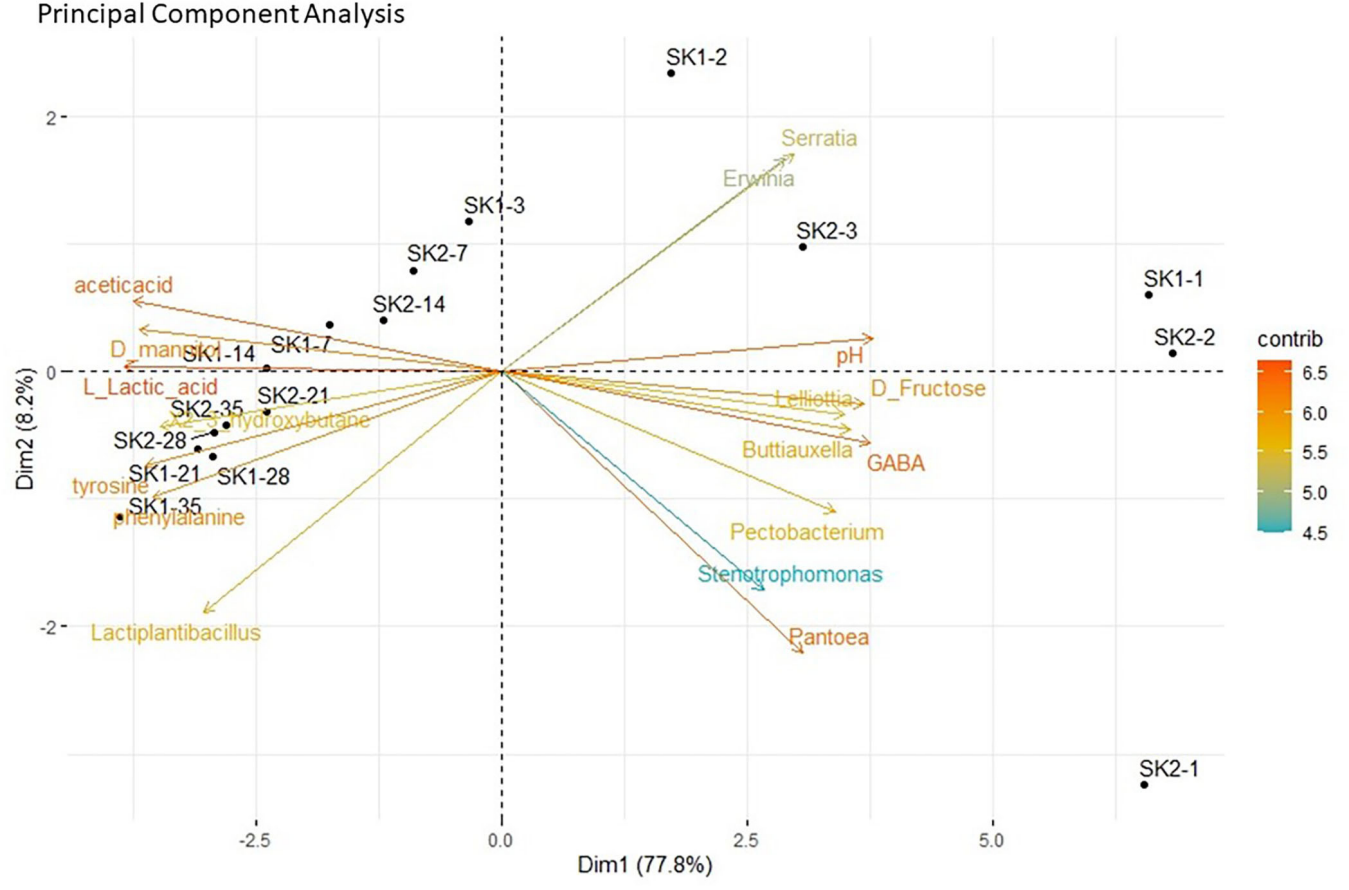
Principal component analysis (PCA) biplot showing correlations between bacterial genera and metabolites at different stages of sauerkraut fermentation. Bacterial genera and metabolites which are spatially together in the same direction along an axis suggest positive correlations; metabolites and genera located in different directions indicate no correlations. The strength of the correlation is shown by the increasing distance from the center of the plot.

The ^1^H-NMR was performed by the Traceability Unit at Fondazione Edmund Mach to quantify microbial metabolites in sauerkraut brine samples from both producers (SK1 and SK2). The results of metabolite quantifications are shown in Figure 3 and Supplementary Table S1 and represent the average of the determinations made in the five tanks. Metabolites that were below the detection limit in 70% of total samples were excluded from further analysis. A total of 29 compounds, including organic acids, amino acids, and sugars, were identified in sauerkraut brine samples over 35 days of fermentation (Supplementary Tables S1, S2). The concentration of organic acids, including lactic, acetic, propionic, butyric, and succinic acids, significantly varied over time (Supplementary Tables S1, S2, Figure 3A—Organic acids). Among SCFAs, acetic acid significantly increased over sauerkraut fermentation, reaching its maximum concentration on day 28 for SK2 (38.98 ± 4.96 mM, mean ± SD) and on day 35 for SK1 (32.04 ± 4.97 mM; Supplementary Tables S1, S2). Similarly, lactic acid significantly increased during the entire fermentation process, both in SK1 and SK2, reaching its maximum concentration on day 35 (SK1: 104.55 ± 10.58 mM; SK2: 67.96 ± 39.46 mM; Supplementary Tables S1, S2). Malic and butyric acids only increased their concentration at the end of sauerkraut fermentation, at days 21 and 28, respectively, and only in SK2. The maximum concentration of both acids was reached on day 35 (Figure 3A—Organic acids). A total of five amino acids, including alanine (Ala), leucine (Leu), phenylalanine (Phe), tyrosine (Tyr), and valine (Val), were identified (Supplementary Table S1, Figure 3B—Amino acids). Aromatic amino acids Phe and Tyr significantly increased during sauerkraut fermentation (SK1: day 1 vs. day 35, Phe *p* < 0.001; Tyr *p* < 0.001; SK2: day 1 vs. day 28, Phe *p* = 0.0047; Tyr *p* = 0.006) reaching their maximum concentration at day 28 for SK2 (Phe = 39.98 ± 2.15 mg/L, Tyr = 47.47 ± 4.51 mg/L) and at day 35 for SK1 (Phe = 56.83 ± 7.16 mg/L, Tyr 56.53 ± 5.19 mg/L). On the other hand, most of the sugars, including D-fructose, α-D-glucose, and β-D-glucose, showed decreasing concentrations over time (Supplementary Table S1, Figure 3C—Sugars). However, only D-fructose changes over time were statistically significant after FDR correction (Supplementary Table S2). In samples from both producers, a significant increase in D-mannitol levels was observed after 7 days of fermentation (Supplementary Tables S1, S2, Figure 3E—Other compounds). Another 13 compounds were found in sauerkraut brine samples, including D-mannose (Supplementary Table S1, Figure 3C—Sugars), acetaldehyde, dimethyl sulfoxide (DMSO), deoxyuridine monophosphate, ethanol, 2,3-butanediol, γ-aminobutyric acid (GABA) (Supplementary Table S1, Figure 3D—Other compounds), and methanol, putrescine, succinamide, succinimide, trimethylamine N-oxide (TMAO), and uracil (Supplementary Table S1, Figure 3E—Other compounds). Diverse patterns were observed for these compounds. Ethanol, 2,3'-butanediol, methanol, putrescine, and uracil significantly increased over time, in both producers. GABA levels strongly decreased after 3 or 7 days of fermentation in both producers (SK1, day 1: 52.80 ± 7.23, day 3: not detected; SK2, day 1: 74.25 ± 14.27, day 7: not detected). Sparse PCA suggests a strong contribution of specific bacterial taxa to the production of fermentation metabolites (Figure 4). PCA biplot shows that the concentration of organic acids, especially lactic and acetic acids, as well as the level of some amino acids (phenylalanine and tyrosine), was correlated with the abundance of *Lactiplantibacillus*, particularly in mature sauerkraut brine (T35 and T28). On the other hand, *Pantoea*, a bacteria with the ability to metabolize toxic isothiocyanates, was observed at the early stages of fermentation (T1, T2) and it seemed to cluster in the same direction as GABA, also detected in sauerkraut brine sampled at day 1 and 2 (Luziatelli et al., 2020; Shukla and Beran, 2020).

### Effect of sauerkraut brine supernatants on intestinal epithelial barrier integrity and inflammation

**Figure 5:**
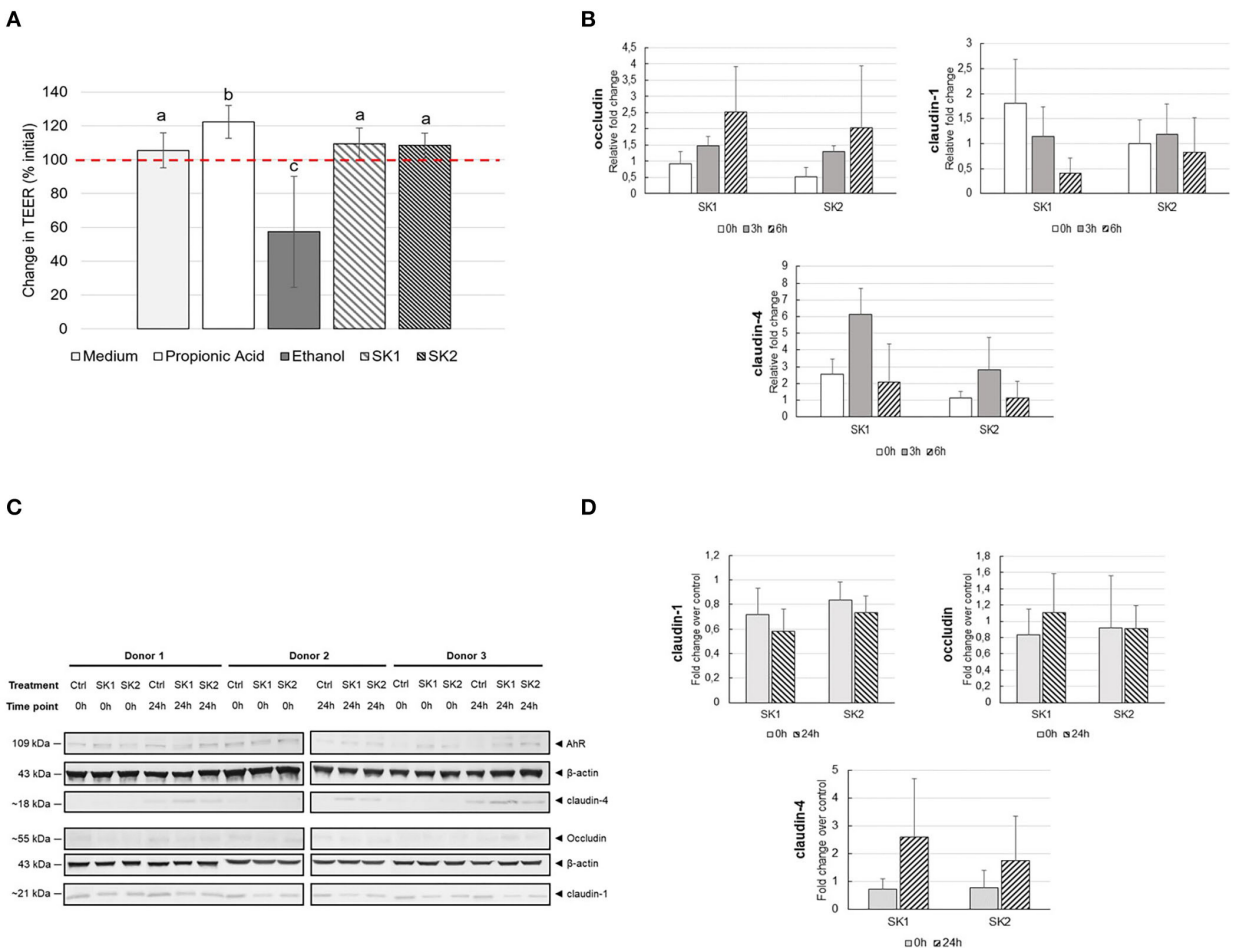
**(A)** Changes in trans-epithelial electrical resistance (TEER) across differentiated Caco-2 monolayers after 24 h of incubation in the presence/absence of 10% SK brine or control medium (TEER_24h_ (Ω cm^2^)/TEER_0h_ (Ω cm^2^) × 100%). The threshold (100%) indicates the baseline TEER value. Different superscript letters indicate statistical significance between test substrates. **(B)** Gene expression of TJ proteins in Caco-2 cells after stimulation with 10% SK brine in Caco-2-PBMC co-culture *in vitro* model. Histograms represent the mean and error bars represent the standard deviation (*n* = 3). **(C)** Quantification of TJ proteins by Western blot. The molecular weight of each protein is described on the left side of the image. β-actin was used as the reference protein. Densitometric analysis by ImageJ software. **(D)** Fold change TJ protein expression normalized to medium control (Ctrl) samples. No significant differences were observed between different treatments and between different time points.

Fermented food consumption was previously suggested to improve immune function and protect against inflammation, possibly due to the ability of microbial fermentation metabolites to tighten gut barrier integrity (Higashikawa et al., 2010; Moroti et al., 2012). The potential of sauerkraut brine to improve gut health was measured, coupling TEER measurement of Caco-2 monolayer before and after 24 h of incubation with 10% sauerkraut brine, and a Caco-2/PBMCs *in vitro* co-culture model, to evaluate both gut epithelial barrier integrity and anti-inflammatory effects of sauerkraut brine. Figure 5 illustrates changes in TEER (Ωcm^2^) expressed as percentage (%) increase or decrease after 24 h of incubation with test substrates, compared to baseline TEER values. As expected, a clear-cut reduction of TEER after 24 h of exposure to ethanol compared with the control medium was observed. An evident TEER improvement after 24 h of exposure to propionic acid, a positive control, was also detected (Ctrl: 105.52 ± 10.38 %; ethanol: 57.36 ± 32.76 %; propionic acid: 122.35 ± 9.64%; *p* = 0.0289 Ctrl vs. ethanol; *p* = 0.0139 Ctrl vs. propionic acid). Twenty-four hours exposure to SK1 and SK2 supernatants showed significantly lower TEER values when compared to positive control and significantly higher values when compared to negative control (SK1: 109.34 ± 9.50%, SK2: 108.44 ± 7.25%; SK1 vs. propionic acid: *p* = 0.0424; SK2 vs. propionic acid: *p* = 0.0168; SK1 vs. ethanol: *p* = 0.0024; SK2 vs. ethanol: *p* = 0.0023). No significant decrease in TEER values was observed after incubation with SK1 or SK2 compared to the control medium. Gene expression of tight junction (TJ) proteins in Caco-2 by RT-PCR was performed to support TEER results (Figure 5). A trend toward increased expression of occludin from 0 to 6 h was observed, although this increase was not statistically significant. No significant changes were observed in claudin-1 and claudin-4 gene expression after incubation with the different treatments. The quantity of TJ proteins in Caco-2 cells was further analyzed by Western blot (Figure 5). No significant enhancement in occludin and claudin-1 protein expression was found after 24 h of incubation with SK1 or SK2. A slight trend toward increased expression of the claudin-4 protein, although not statistically significant, was observed 24 h after incubation with SK1 and SK2 in all experimental replicates (Figure 5).

**Figure 6:**
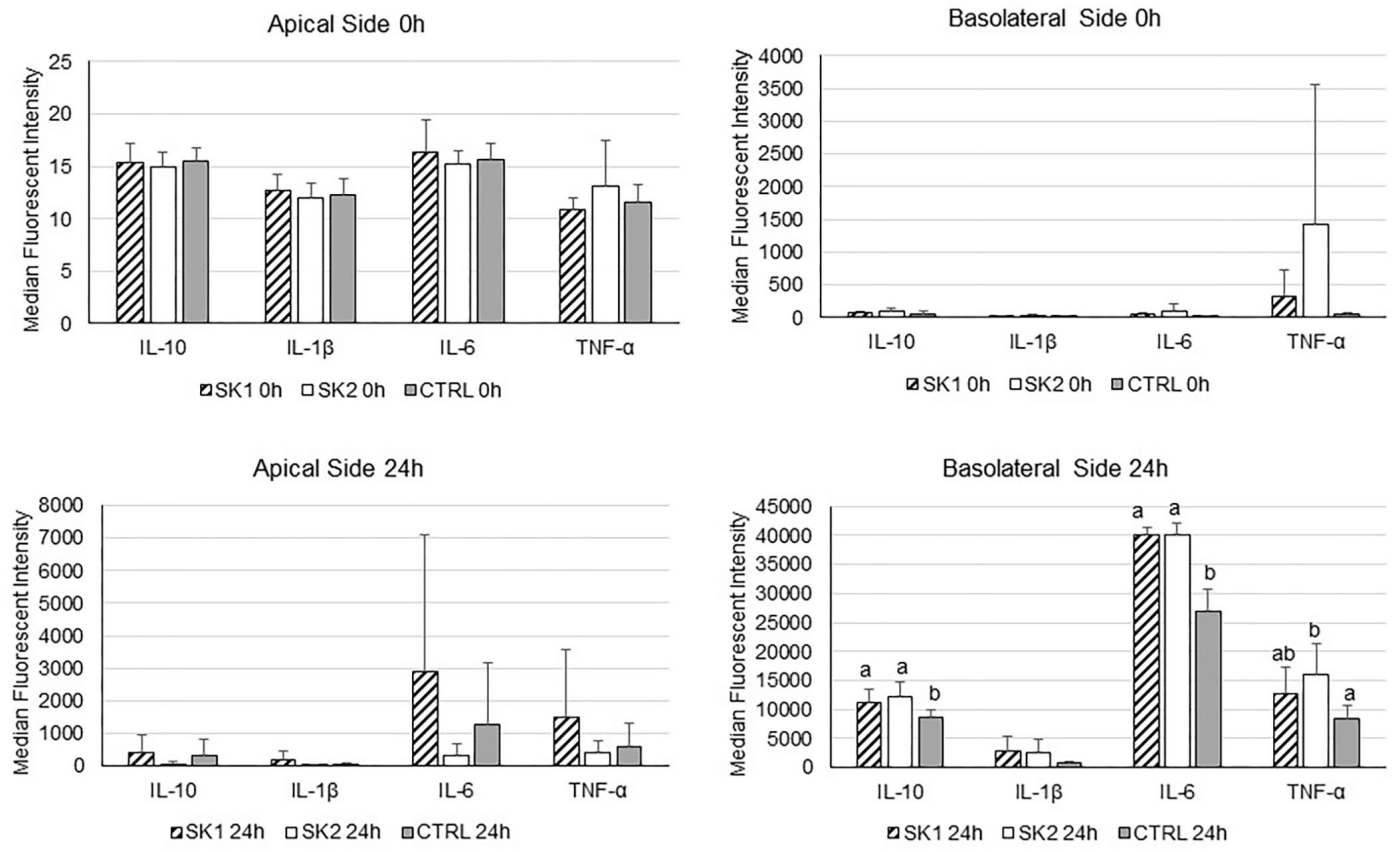
IL-10, IL-1β, IL-6, and TNF-α secretion by co-cultured Caco-2 (apical side) and PBMCs (basolateral side) under LPS stimulation at baseline (0h) and after 24 h of incubation with sauerkraut brine (SK1 and SK2) or control cell culture medium (CTRL). Bar plots represent median values of median fluorescent intensity (MFI) of the Magpix^®^ signal, and the error bars represent standard deviation (*n* = 3). Note the different scales on the y-axis. Different superscript letters indicate statistically significant differences within each cytokine.

Cytokine concentrations were measured in upper and basolateral supernatants collected for the co-culture experiments at baseline and after 24 h of incubation with sauerkraut fermentation brine (Figure 6). In general, the data show high variability in cytokine secretion, probably due to the inter-individual variability of PBMCs obtained from different healthy donors, as already suggested (Katial et al., 1998). At baseline, there was no significant difference in cytokine concentration between treatments both in the basolateral (PBMCs) and in the upper side (Caco-2). As expected, the LPS stimulus induced a dramatic increase in all cytokine levels, both on the upper and basolateral sides, as shown in 24-h panels (Figure 6). However, no differences were observed when comparing sauerkraut brine and control for any of the cytokines secreted by Caco-2 in the upper supernatants. On the other hand, statistically significant differences between sauerkraut brine and control were observed in the cytokine secreted by PBMC in the basolateral supernatants. SK1 and SK2 stimulated PBMCs to produce IL-6 (SK1: 40,190.10 ± 1,170.24 MFI; SK2: 40,136.50 ± 2,001.52 MFI; Ctrl: 26,971.2 ± 3,660.37 MFI; mean ± SD; SK1 vs. Ctrl, *p* < 0.0001; SK2 vs. Ctrl, *p* < 0.0001) and IL-10 (SK1: 11,085.25 ± 2,256.28; SK2: 12,115.3 ± 2,525.93; Ctrl: 8,656.67 ± 1,357.85 MFI, SK1 vs. Ctrl, *p* = 0.047; SK2 vs. Ctrl, *p* = 0.014), while only SK2 induced increased production of TNF-α (SK2: 16,092.90 ± 5,173.35 MFI; Ctrl: 8,338.58 ± 2,328.42 MFI; SK2 vs. Ctrl, *p* = 0.0074). Cytokine gene expression in PBMCs was investigated by RT-PCR. No significant changes were observed in IL-10 and IL-6 expression after 3 h and 6 h of incubation with sauerkraut brine.

## Discussion

The growing interest in the potential health effects of fermented foods, including traditionally fermented plant foods, is generating interest in characterizing their associated microbiota and providing scientific evidence for their ability to influence human immune function. In this work, the successional development of sauerkraut microbiota and its metabolic output over the course of spontaneous fermentation were characterized. The potential of sauerkraut brine to modulate gut permeability and regulate intestinal inflammatory response was tested, using an *in vitro* co-culture model of the intestinal epithelial barrier (Caco-2 monolayer) and circulating immune cells (PBMCs).

The temperature increased over the first week of fermentation, reaching a maximum average value by day 10 (SK1: 22.42 ± 0.27°C, SK2: 16.15 ± 0.30°C, mean ± SD), and then decreased to approach environmental/ambient temperature, consistent with previous reports on similar fermented foods (Vaughn, 1927). The increase in temperature occurred concomitantly with a reduction in pH between days 1 and 14 due to the production of organic acids by fermentative bacteria, according to ^1^H-NMR analyses.

As the fermentation proceeded, the same changes in sauerkraut brine microbiota in all five tanks of both producers were observed, with the microbial diversity gradually decreasing and LAB becoming the dominant bacteria, as confirmed by the 16S rRNA Illumina sequencing. At the early stages of fermentation, the microbial composition included microorganisms from cabbage itself, such as *Pantoea*, and also *Enterobacter, Serratia, Pseudomonas, Pectobacterium, Lelliottia, Buttiauxella*, and *Acinetobacter*. These findings are in concordance with the previous studies, suggesting that the presence of *Pseudomonadaceae, Enterobacteriaceae*, and *Moraxellaceae* mainly derives not only from the raw vegetables but also from the surface of the fermentation environment, including the floor. On the other hand, *Leuconostocaceae* and *Lactobacillaceae*, which increased over time, were previously suggested to colonize surfaces of fermentation tanks and the environment (including floors, handles, and tools surfaces; Einson et al., 2018). However, in this study, samples from the environment where fermentation was carried out were not analyzed. When considering the relative abundance of genera, a clear difference was observed between the two sauerkraut producers. Although at later time points the most abundant genus was *Lactiplantibacillus*, in one of the two producers (SK1), *Leuconostoc* and *Paucilactobacillus* were also highly abundant. In SK2, a lower abundance of *Leuconostoc* and a greater diversity of Lactobacillaceae (*Levilactobacillus, Pediococcus, Secundilactobacillus*, and *Lentilactobacillus*) was detected. These differences in microbial diversity may be the result of spontaneous fermentation, with different plant/cabbage-associated starting inocula, cross-contamination from the production plant and equipment, and the food production environment possibly contributing to different microbial compositions at the early stages of fermentation. However, the drop in species diversity and the emergence of a small number of dominant strains, evident in this study using both culture-dependent and culture-independent methods, highlights the selective nature of the fermentation process.

During the first days, the fermentation was mainly carried out by *Leuconostoc* species, which gradually increased until day 7. A change in the microbial composition was observed on day 14, with a decrease in the abundance of *Leuconostoc* and a concomitant gradual increase in *Lactiplantibacillus* spp, together with *Paucilactibacillus* spp. or with *Pediococcus* spp. and *Levilactobacillus* spp. While bacterial α-diversity indices Chao1 and observed OTUs significantly decreased over the fermentation process, thus indicating lower bacterial richness with the predominance of LAB taxa, the Shannon diversity index showed a progressive increase in α-diversity from day 7 (SK1) or day 14 (SK2) until day 35, thus reflecting increased evenness amongst the LAB actively involved in the fermentation (Zabat et al., 2018). 16S rRNA PCoA analysis showed a clear clustering of sauerkraut brine microbiota according to fermentation time point.

After 35 days of fermentation, LAB *Lactiplantibacillus, Leuconostoc, Pediococcus, Levilactobacillus, Paucilactobacillus*, and *Secundilactobacillus* were the predominant genera. These findings are consistent with previous descriptions of sauerkraut microbiota, which used both culture-dependent (Shukla and Beran, 2020) and culture-independent methods (Plengvidhya et al., 2007; Zabat et al., 2018; Yang et al., 2020) and identified *Leuconostoc, Lactobacillus*, and *Pediococcus* as the primary microorganisms in sauerkraut fermentation from both producers.

The pH, ^1^H-NMR, and 16S rRNA results confirm that the levels of acidity, the concentration of lactic acid, and the abundance of LAB are strictly correlated and show an increasing trend until the end of fermentation. Besides being the most important bacteria involved in the fermentation process, LAB are known for their possible probiotic properties (Plengvidhya et al., 2007; Marco et al., 2017; Dimidi et al., 2019). Sequencing identification of isolates collected from each producer fermentation revealed the specificity of fermentative LAB at the early stages of fermentation, with *Leuconostoc mesenteroides, Leuconostoc citreum*, and *Lactococcus lactis* being the most prevalent species in SK1, and *Leuconostoc pseudomesenteroides, L. mesenteroides, Lactiplanctibacillus pentosus*, and *Lactiplanctibacillus plantarum* in SK2. However, this changed over the course of the fermentation, with the emergence of common species toward the end of the fermentation process. *L. plantarum, L. paraplantarum*, and *L. brevis* were the only species to be efficiently and consistently isolated from sauerkraut brine samples for both producers. These results confirm the intrinsic resistance of LAB to acidic environments, identify the species responsible for driving the sauerkraut fermentation, and also how the shared environmental pressures select a particular species of LAB during fermentation. *Leuconostoc* and *Paucilactobacillus* were detected by metataxonomic analysis at the end of fermentation in SK1 but were not isolated on MRS agar. This highlights the limitation of selective culture media where only the dominant members of a target microbiota may be identified.

In the present study, ^1^H-NMR analyses were used for high-throughput and highly reproducible (Nicholson et al., 1999) characterization of metabolites in sauerkraut brine samples over time. An accumulation of organic acids was observed over the fermentation process, with significant production of acetic and lactic acids. Butyric and propionic acids significantly increased only in SK2 producers reaching the highest levels at day 35, while their concentration did not vary in SK1. This is consistent with the greater microbial diversity observed in the fermentations of producer 2 (SK2). The increase in organic acids was parallel to the decrease in pH values. Together with acidification, proteolysis is considered a key feature of food fermentation, since it influences the production of flavor compounds and potentially bioactive compounds like GABA, and it also increases polypeptide digestibility (Zhao et al., 2016; Rizzello et al., 2019). In this study, a significant increase in the concentration of aromatic amino acids Phe and Tyr was observed, which may contribute to the typical aroma and flavor of fermented cabbage, as previously suggested (Jagannath et al., 2012; Harth et al., 2016). During the fermentation process, a significant increase in the hydrophobic amino acid leucine was detected, which has been suggested to be responsible for the bitter taste in different fermented foods (Baek et al., 2010). GABA, a non-protein amino acid that serves as the major inhibitory neurotransmitter in the brain and spinal cord (Kondziella, 2017), decreased in concentration during the fermentation process. This is consistent with previous studies, where GABA was converted to other metabolites, such as succinate during fermentation (Shelp et al., 1999; Baek et al., 2010). GABA degradation to succinate (also known as the GABA shunt) mainly occurs by the action of the enzyme GABA transaminase, which catalyzes the reaction using either pyruvate or alpha-ketoglutarate as amino receptor (Berg et al., 2007). As recently observed by Yogeswara et al. (2021) who cultivated a strain of *Lactobacillus brevis* isolated from Indonesian fermented foods (fermented soybeans, *growol, gatot, tempeh*, and *bekasam*), it is suggested that GABA could be used as a bacterial nutrient after 3 (SK1) or 7 (SK2) days of sauerkraut fermentation. Also, over 35 days of fermentation, a significant increase in the concentration of uracil was observed in the sauerkraut brine of both producers. Uracil has been previously identified as a metabolic product of LAB fermentation, especially of *L. plantarum* metabolism (Liu et al., 2015). Again, this is in agreement with the microbiological succession described in sauerkraut fermentations and confirms that changes in metabolites are key indicators of the activity of specific bacteria during sauerkraut fermentation. For example, D-fructose consumption together with the increase in mannitol concentration may be attributed to the metabolic activity of *Leuconostoc* species. *Leuconostoc* was previously shown to utilize fructose as an alternative electron acceptor to produce mannitol (Harth et al., 2016).

The Caco-2 cell monolayer on a trans-well system was used to test whether sauerkraut brine could improve intestinal epithelial barrier integrity. Sauerkraut brine from both SK1 and SK2 contributed to the maintenance of physiological TEER values, which were comparable to that of the medium, higher than the negative control, ethanol, but significantly lower than the positive control, propionic acid. Gene and protein expression of TJ in Caco-2 cells did not reveal significant changes after incubation with sauerkraut brine, thus confirming TEER results. In our work, no changes were observed in claudin-1, claudin-4, occludin mRNA, or protein expression in Caco-2. Claudin-1, claudin-4, and occludin play a key role in the maintenance of intestinal barrier integrity (Gao et al., 2018; Wu et al., 2020). Downregulation of claudin-1 and occludin was previously observed in Caco-2 cells after LPS stimulation, together with increased gut permeability (Prasad et al., 2005). Also, downregulation of claudin-4 and increased paracellular permeability were observed after TNF-α inflammatory stimulus in another study that employed T84 cell monolayers (Prasad et al., 2005). Studies on *in vitro* intestinal epithelial integrity highlight the role of TJ proteins in gut barrier function maintenance, and suggest an impairment of gut barrier function during inflammation. To further investigate the possible immune modulatory effects of sauerkraut brine, the monolayer of Caco-2 cells co-cultivated with underlying PBMCs and stimulated with LPS was employed. No significant changes in the mRNA expression of inflammatory interleukin IL-6 and anti-inflammatory IL-10 in PBMCs after 3 and 6 h of incubation with sauerkraut brine were found. However, cytokine quantification in basolateral supernatants revealed a significant increase in IL-6, IL-10, and TNF-α levels after 24 h of incubation with sauerkraut brine. IL-6 and TNF-α are pro-inflammatory cytokines typically released by various cell types, including intestinal mucosal immune cells and PBMC, during LPS stimulation (Meng and Lowell, 1997; Zhou et al., 2016; Dittel et al., 2020), while IL-10 is an anti-inflammatory mediator crucial in maintaining an adequate balance of the inflammatory response and is involved in resolving inflammation after an inflammatory trigger (Latorre et al., 2014). The results from the present study indicated that sauerkraut brine has the potential to improve immune function, supporting an appropriate immune response to LPS challenge (increased IL-6 and TNF-α) and importantly, tempering the inflammatory response through the production of anti-inflammatory IL-10. The fact that sauerkraut brine induced IL-10 production suggests that it might promote the resolution of inflammation and re-establishment of normal inflammatory status after an inflammatory challenge. This is important for normal or optimal immune function both within the gastrointestinal tract and at exo-gastrinal sites, since unresolved inflammation has been identified as a risk factor for metabolic derangement associated with cardiovascular disease and type 2 diabetes, and may also play an etiological role in autoimmune diseases. In the present study, the anti-inflammatory role of sauerkraut brine may be driven by acetic and lactic acids, both present at high concentrations after 35 days of fermentation, and both identified as immune response mediators due to their ability to bind G protein-coupled receptors (Okada et al., 2013; Parada Venegas et al., 2019). Latham et al. (2012) highlighted the role of lactic acid in inhibiting the histone deacetylases HDAC11, a suppressor of IL-10 expression, thus suggesting that lactic acid may act as a transcriptional regulator, linking microbial metabolism to immunomodulation. In our experiments, no changes in IL-10 gene expression after 3 and 6 h of incubation with sauerkraut brine were observed, but higher concentrations of IL-10 protein were detected in PBMC supernatants after 24 h of incubation with SK brine in the presence of an inflammatory stimulus.

In this study, the spontaneous fermentation of white cabbage was characterized. The successional development of the sauerkraut-associated microbiota was evaluated using both culture-dependent and culture-independent methodologies, and the production of different microbial metabolites, including organic acids, amino acids, amines, and sugars, was measured. This work confirms the potential of the fermentation process to select a food type-specific microbiome, one which has the potential to influence both the technological and sensory characteristics of the food product *via* metabolite production. The combined microbial composition and metabolite profile may also play a role in some of the gastrointestinal health effects of sauerkraut. This study is one of the few studies that demonstrate experimentally that sauerkraut brine modulates the gut barrier and can help regulate intestinal immune function (Raak et al., 2014). Further *in vivo* studies will be necessary to confirm these observations. Studies that characterize and preserve the microbial natural biodiversity of traditional fermented plant foods are important contributions to protecting food-associated microbiota, offering new solutions for improving human health and adding value to traditional food production processes.

## Data availability statement

The data presented in the study are deposited in the EBI repository (https://www.ebi.ac.uk/metagenomics/), accession number PRJEB51696.

## Author contributions

TW, LBe, CP, and FF contributed to the conception and design of the study. GG, TW, GM, LBe, and FF made the sampling. GG, TW, GM, PS, and FF performed the experiments. GG, TW, PS, EF, LBo, and FF analyzed the data. GG wrote the first draft of the manuscript. All authors contributed to the writing, revision, and approval of the submitted manuscript.

## Funding

This work was supported by the EUREGIO: Environment, Food and Health (EUREGIO-EFH) project (deliberazione della Giunta Provinciale di Trento n. 2406, 20.12.2016) funded by Fondazione Mach e GECT Euregio Tirolo-Alto Adige-Trentino. EUREGIO-EFH funded the Ph.D. scholarship of GG and paid for publication fees. The authors declare that this study also received funding from the Autonomous Province of Trento, with EU co-financing (FRUITOMICS, FESR 2014–2020 Program of the Autonomous Province of Trento, Italy). The funder was not involved in the study design, collection, analysis, interpretation of data, the writing of this article, or the decision to submit it for publication.

## Conflict of interest

The authors declare that the research was conducted in the absence of any commercial or financial relationships that could be construed as a potential conflict of interest.

## Publisher's note

All claims expressed in this article are solely those of the authors and do not necessarily represent those of their affiliated organizations, or those of the publisher, the editors and the reviewers. Any product that may be evaluated in this article, or claim that may be made by its manufacturer, is not guaranteed or endorsed by the publisher.

## Supplementary material

Supplementary Figure S1 Schematic illustration of *in vitro* model of the intestinal epithelium. Human adenocarcinoma cells (Caco-2) were grown to confluence, and then co-cultured with peripheral blood mononuclear cells (PBMCs) to mimic the intestinal epithelial barrier. Trans-epithelial electrical resistance (TEER) was measured after 24 h of incubation with sauerkraut fermentation brine as an index of intestinal integrity. The potential anti-inflammatory effect of sauerkraut brine was evaluated by measuring cytokine release into the basolateral and apical side upon PBMC exposure to lipopolysaccharide (LPS) for 24 h, following 2 h of pre-incubation in the presence or absence of 10% sauerkraut water. Tight junction (TJ) proteins in Caco-2 cells were measured by Western blot. Gene expression analysis of cytokines and TJ proteins was also performed.

Supplementary Table S1 Metabolite concentration (mg/L) in sauerkraut brine quantified by ^1^H-NMR. Data represent the mean and standard deviation (SD) after 1, 2, 3, 7, 14, 21, 28, and 35 days of fermentation. Producer 1 (SK1) **(A)** and producer 2 (SK2) **(B)**. DMSO, dimethyl sulfoxide; dUMP, deoxyuridine monophosphate; GABA, γ -aminobutyric acid.

Supplementary Table S2 *P*-values of statistically significant differences in metabolite concentration in sauerkraut brine from SK1 **(A)** and from SK2 **(B)** after Kruskal–Wallis test, followed by the *post-hoc* Dunn's test with Benjamini–Hochberg false discovery rate (FDR) *p*-value correction.
