# Supplementary figures and images for: Microbial and metabolic characterization of organic artisanal sauerkraut fermentation and study of gut health-promoting properties of sauerkraut brine
Source: Front Microbiol. 2022 Oct 13;13:929738. doi: 10.3389/fmicb.2022.929738 (PMC9606823; doi:10.3389/fmicb.2022.929738)

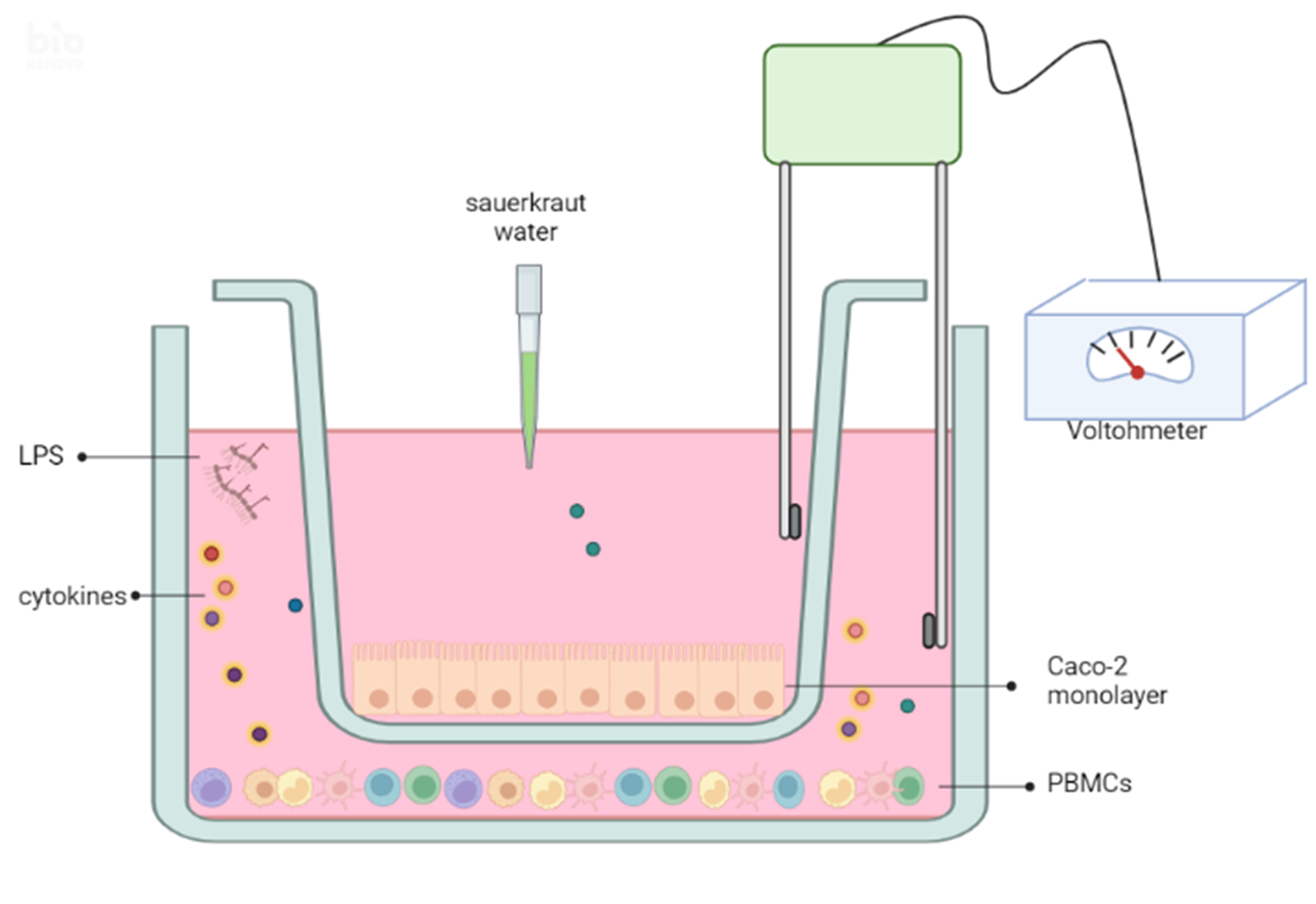

Supplement: Supplementary Figure S1 — Schematic illustration of in vitro model of the intestinal epithelium. Human adenocarcinoma cells (Caco-2) were grown to confluence, and then co-cultured with peripheral blood mononuclear cells (PBMCs) to mimic the intestinal epithelial barrier. Trans-epithelial electrical resistance (TEER) was measured after 24 h of incubation with sauerkraut fermentation brine as an index of intestinal integrity. The potential anti-inflammatory effect of sauerkraut brine was evaluated by measuring cytokine release into the basolateral and apical side upon PBMC exposure to lipopolysaccharide (LPS) for 24 h, following 2 h of pre-incubation in the presence or absence of 10% sauerkraut water. Tight junction (TJ) proteins in Caco-2 cells were measured by Western blot. Gene expression analysis of cytokines and TJ proteins was also performed. [file Image_1.PNG]
