# Supplementary material for: Microbial and metabolic characterization of organic artisanal sauerkraut fermentation and study of gut health-promoting properties of sauerkraut brine
Source: Front Microbiol. 2022 Oct 13;13:929738. doi: 10.3389/fmicb.2022.929738 (PMC9606823; doi:10.3389/fmicb.2022.929738)
Supplement: Supplementary Table S1 — Metabolite concentration (mg/L) in sauerkraut brine quantified by 1H-NMR. Data represent the mean and standard deviation (SD) after 1, 2, 3, 7, 14, 21, 28, and 35 days of fermentation. Producer 1 (SK1) (A) and producer 2 (SK2) (B). DMSO, dimethyl sulfoxide; dUMP, deoxyuridine monophosphate; GABA, γ -aminobutyric acid. [file Table_1.docx]

## Supplementary material

**Table S1.** Metabolites concentration (mg/L) in sauerkraut brine quantified by H-NMR. Data represent the mean and standard deviation (SD) after 1, 2, 3, 7, 14, 21, 28 and 35 days of fermentation. Producer 1 (SK1) (**A**), producer 2 (SK2) (**B**). DMSO, dimethyl sulfoxide; dUMP, deoxyuridine monophosphate; GABA, γ -aminobutyric acid.

| **A. SK1** | **Day 1** | **Day 2** | **Day 3** | **Day 7** | **Day 14** | **Day 21** | **Day 28** | **Day 35** |
| --- | --- | --- | --- | --- | --- | --- | --- | --- |
| **Organic acids** |  |  |  |  |  |  |  |  |
| Acetic acid |  |  |  |  |  |  |  |  |
| mean | 0.00 | 441.25 | 944.35 | 1281.35 | 1568.03 | 1767.27 | 1610.06 | 1923.64 |
| SD | 0.00 | 339.46 | 472.53 | 784.27 | 287.08 | 562.87 | 546.43 | 298.16 |
| Butyric acid |  |  |  |  |  |  |  |  |
| mean | 0.00 | 0.00 | 0.00 | 0.00 | 0.00 | 0.00 | 0.00 | 0.00 |
| SD | 0.00 | 0.00 | 0.00 | 0.00 | 0.00 | 0.00 | 0.00 | 0.00 |
| Formic acid |  |  |  |  |  |  |  |  |
| mean | 3.87 | 4.88 | 11.88 | 1.93 | 1.84 | 3.50 | 3.04 | 3.77 |
| SD | 2.17 | 3.97 | 12.65 | 1.40 | 0.46 | 1.40 | 2.02 | 0.60 |
| Lactic acid |  |  |  |  |  |  |  |  |
| mean | 17.66 | 650.02 | 1397.50 | 3634.37 | 7148.75 | 8713.62 | 4228.90 | 9418.22 |
| SD | 3.16 | 348.11 | 579.28 | 1994.91 | 1018.56 | 2193.76 | 3939.70 | 953.14 |
| Malic acid |  |  |  |  |  |  |  |  |
| mean | 0.00 | 0.00 | 0.00 | 0.00 | 0.00 | 0.00 | 0.00 | 0.00 |
| SD | 0.00 | 0.00 | 0.00 | 0.00 | 0.00 | 0.00 | 0.00 | 0.00 |
| Propionic acid |  |  |  |  |  |  |  |  |
| mean | 7.62 | 8.75 | 10.73 | 11.86 | 14.97 | 14.68 | 12.14 | 13.84 |
| SD | 1.61 | 2.32 | 3.54 | 5.79 | 1.61 | 2.14 | 8.02 | 8.01 |
| Succinic acid |  |  |  |  |  |  |  |  |
| mean | 15.12 | 32.83 | 54.32 | 24.80 | 15.12 | 17.71 | 18.66 | 25.27 |
| SD | 8.20 | 25.78 | 25.68 | 7.70 | 0.99 | 3.23 | 3.95 | 3.19 |
| **Amino acids** |  |  |  |  |  |  |  |  |
| Alanine |  |  |  |  |  |  |  |  |
| mean | 95.33 | 122.41 | 156.09 | 170.16 | 189.05 | 154.48 | 86.42 | 155.19 |
| SD | 15.50 | 55.83 | 65.80 | 97.64 | 20.88 | 83.80 | 118.79 | 142.26 |
| Leucine |  |  |  |  |  |  |  |  |
| mean | 20.99 | 26.23 | 34.37 | 46.96 | 62.96 | 80.01 | 82.64 | 109.92 |
| SD | 2.78 | 11.05 | 14.35 | 25.96 | 11.09 | 30.25 | 33.34 | 14.71 |
| Phenylalanine |  |  |  |  |  |  |  |  |
| mean | 5.62 | 13.88 | 19.16 | 23.79 | 30.06 | 40.97 | 41.63 | 56.83 |
| SD | 3.98 | 7.07 | 7.80 | 13.45 | 6.11 | 15.90 | 16.16 | 7.16 |
| Tyrosine |  |  |  |  |  |  |  |  |
| mean | 0.00 | 19.21 | 28.99 | 25.37 | 34.79 | 46.02 | 42.76 | 56.53 |
| SD | 0.00 | 12.66 | 15.16 | 16.56 | 8.82 | 13.47 | 15.03 | 5.19 |
| Valine |  |  |  |  |  |  |  |  |
| mean | 32.80 | 40.77 | 57.87 | 61.39 | 65.37 | 79.19 | 83.41 | 102.15 |
| SD | 4.76 | 21.37 | 25.26 | 38.47 | 7.24 | 21.34 | 23.23 | 6.80 |
| **Sugars** |  |  |  |  |  |  |  |  |
| α -D-Glucose |  |  |  |  |  |  |  |  |
| mean | 2201.56 | 2584.58 | 3093.71 | 2288.03 | 1474.43 | 1409.93 | 1398.76 | 1799.80 |
| SD | 301.24 | 1567.87 | 1452.38 | 1307.72 | 93.40 | 293.67 | 504.74 | 151.32 |
| β-D-Glucose |  |  |  |  |  |  |  |  |
| mean | 3214.05 | 3800.30 | 4635.16 | 3616.89 | 2385.68 | 2343.88 | 2149.31 | 2869.59 |
| SD | 448.57 | 2512.57 | 2289.34 | 2004.13 | 132.50 | 578.54 | 786.52 | 227.60 |
| D-Fructose |  |  |  |  |  |  |  |  |
| mean | 1122.40 | 688.21 | 398.15 | 141.61 | 176.56 | 191.69 | 172.59 | 265.56 |
| SD | 154.91 | 339.92 | 108.01 | 71.13 | 32.97 | 59.68 | 74.62 | 111.22 |
| D-Mannose |  |  |  |  |  |  |  |  |
| mean | 0.00 | 0.00 | 0.00 | 19.10 | 0.00 | 0.00 | 16.21 | 0.00 |
| SD | 0.00 | 0.00 | 0.00 | 14.39 | 0.00 | 0.00 | 22.28 | 0.00 |
| **Other compounds** |  |  |  |  |  |  |  |  |
| 2,3-Butanediol |  |  |  |  |  |  |  |  |
| mean | 0.00 | 53.53 | 157.35 | 213.94 | 262.97 | 337.41 | 329.12 | 380.85 |
| SD | 0.00 | 92.18 | 190.09 | 238.50 | 179.05 | 241.85 | 232.92 | 178.00 |
| Acetaldehyde |  |  |  |  |  |  |  |  |
| mean | 0.00 | 0.00 | 0.00 | 0.00 | 2.82 | 3.52 | 3.17 | 2.20 |
| SD | 0.00 | 0.00 | 0.00 | 0.00 | 2.67 | 0.93 | 0.48 | 1.28 |
| DMSO |  |  |  |  |  |  |  |  |
| mean | 0.00 | 8.28 | 11.88 | 4.69 | 3.91 | 5.16 | 4.84 | 6.56 |
| SD | 0.00 | 3.34 | 8.18 | 3.62 | 0.78 | 1.31 | 1.40 | 0.89 |
| D-Mannitol |  |  |  |  |  |  |  |  |
| mean | 0.00 | 2418.49 | 4712.37 | 5516.47 | 6587.63 | 7293.36 | 6359.92 | 7734.94 |
| SD | 0.00 | 1672.67 | 2531.59 | 3430.65 | 1341.11 | 2287.44 | 2078.10 | 1393.15 |
| dUMP |  |  |  |  |  |  |  |  |
| mean | 0.00 | 0.00 | 0.00 | 0.00 | 0.00 | 0.00 | 0.00 | 0.00 |
| SD | 0.00 | 0.00 | 0.00 | 0.00 | 0.00 | 0.00 | 0.00 | 0.00 |
| Ethanol |  |  |  |  |  |  |  |  |
| mean | 607.21 | 768.34 | 927.90 | 1023.51 | 1275.55 | 1385.11 | 1308.00 | 1541.07 |
| SD | 123.49 | 243.38 | 339.28 | 465.98 | 155.13 | 333.47 | 404.74 | 150.73 |
| GABA |  |  |  |  |  |  |  |  |
| mean | 52.80 | 25.16 | 0.00 | 0.00 | 0.00 | 0.00 | 0.00 | 0.00 |
| SD | 7.23 | 34.46 | 0.00 | 0.00 | 0.00 | 0.00 | 0.00 | 0.00 |
| Methanol |  |  |  |  |  |  |  |  |
| mean | 29.86 | 35.12 | 42.29 | 52.23 | 79.65 | 131.94 | 136.30 | 172.95 |
| SD | 3.67 | 14.79 | 19.12 | 30.50 | 16.73 | 68.09 | 68.99 | 33.22 |
| Putrescine |  |  |  |  |  |  |  |  |
| mean | 0.00 | 0.00 | 0.00 | 30.32 | 64.35 | 87.44 | 81.45 | 98.73 |
| SD | 0.00 | 0.00 | 0.00 | 23.94 | 46.21 | 69.12 | 65.41 | 42.75 |
| Succinamide |  |  |  |  |  |  |  |  |
| mean | 0.00 | 83.90 | 96.45 | 12.78 | 20.68 | 19.52 | 16.97 | 26.73 |
| SD | 0.00 | 76.90 | 65.27 | 11.82 | 8.36 | 7.59 | 7.91 | 9.00 |
| Succinimide |  |  |  |  |  |  |  |  |
| mean | 4.16 | 12.29 | 12.09 | 2.58 | 2.38 | 3.57 | 3.57 | 3.37 |
| SD | 0.83 | 7.82 | 9.67 | 1.13 | 0.54 | 2.49 | 2.58 | 0.54 |
| Trimethylamine N-oxide |  |  |  |  |  |  |  |  |
| mean | 26.89 | 32.74 | 40.85 | 36.80 | 41.61 | 47.76 | 44.46 | 54.67 |
| SD | 2.98 | 15.33 | 17.54 | 18.56 | 5.22 | 12.80 | 14.08 | 3.92 |
| Uracil |  |  |  |  |  |  |  |  |
| mean | 0.00 | 4.26 | 5.38 | 3.36 | 4.48 | 6.95 | 7.17 | 8.74 |
| SD | 0.00 | 2.01 | 2.56 | 2.63 | 1.37 | 3.49 | 3.60 | 2.79 |
| **B. SK2** | **Day 1** | **Day 2** | **Day 3** | **Day 7** | **Day 14** | **Day 21** | **Day 28** | **Day 35** |
| **Organic acids** |  |  |  |  |  |  |  |  |
| Acetic acid |  |  |  |  |  |  |  |  |
| mean | 0.00 | 75.66 | 522.56 | 1873.56 | 1643.09 | 2037.38 | 2480.19 | 2340.99 |
| SD | 0.00 | 62.99 | 477.37 | 148.62 | 551.08 | 522.57 | 214.00 | 297.64 |
| Butyric acid |  |  |  |  |  |  |  |  |
| mean | 0.00 | 0.00 | 0.00 | 0.00 | 0.00 | 0.00 | 0.00 | 99.04 |
| SD | 0.00 | 0.00 | 0.00 | 0.00 | 0.00 | 0.00 | 0.00 | 57.79 |
| Formic acid |  |  |  |  |  |  |  |  |
| mean | 1.66 | 1.20 | 5.89 | 4.42 | 10.59 | 2.30 | 2.58 | 1.66 |
| SD | 1.06 | 0.77 | 7.66 | 6.94 | 8.67 | 0.65 | 1.51 | 0.95 |
| Lactic acid |  |  |  |  |  |  |  |  |
| mean | 9.91 | 29.91 | 904.58 | 3003.45 | 2388.74 | 5211.31 | 7255.58 | 6121.66 |
| SD | 13.02 | 33.95 | 838.63 | 220.67 | 1777.28 | 1778.69 | 1229.59 | 3554.43 |
| Malic acid |  |  |  |  |  |  |  |  |
| mean | 108.07 | 85.81 | 40.76 | 56.05 | 75.35 | 270.57 | 893.51 | 1370.30 |
| SD | 55.81 | 20.38 | 17.75 | 8.91 | 62.10 | 464.02 | 1542.25 | 1720.16 |
| Propionic acid |  |  |  |  |  |  |  |  |
| mean | 3.67 | 2.54 | 9.04 | 8.19 | 10.73 | 22.87 | 45.18 | 42.92 |
| SD | 2.75 | 3.52 | 2.57 | 7.56 | 3.54 | 11.49 | 34.25 | 30.00 |
| Succinic acid |  |  |  |  |  |  |  |  |
| mean | 38.50 | 38.26 | 49.36 | 78.65 | 56.21 | 34.95 | 14.64 | 13.46 |
| SD | 22.83 | 18.79 | 42.46 | 7.63 | 18.44 | 10.25 | 3.88 | 2.72 |
| **Amino acids** |  |  |  |  |  |  |  |  |
| Alanine |  |  |  |  |  |  |  |  |
| mean | 124.73 | 130.07 | 144.86 | 209.18 | 138.27 | 225.22 | 343.71 | 264.60 |
| SD | 29.33 | 20.64 | 38.73 | 18.91 | 98.29 | 85.14 | 72.22 | 150.65 |
| Leucine |  |  |  |  |  |  |  |  |
| mean | 35.94 | 39.35 | 44.86 | 63.49 | 60.86 | 76.87 | 98.90 | 87.36 |
| SD | 8.63 | 8.55 | 11.71 | 4.60 | 18.82 | 24.50 | 12.33 | 8.01 |
| Phenylalanine |  |  |  |  |  |  |  |  |
| mean | 8.92 | 8.92 | 15.86 | 31.06 | 30.06 | 36.01 | 43.61 | 39.98 |
| SD | 9.95 | 10.22 | 7.44 | 3.39 | 10.86 | 10.21 | 4.76 | 2.15 |
| Tyrosine |  |  |  |  |  |  |  |  |
| mean | 9.42 | 13.05 | 18.84 | 31.16 | 32.61 | 44.57 | 48.56 | 47.47 |
| SD | 7.06 | 4.69 | 3.97 | 11.13 | 9.84 | 12.98 | 7.73 | 4.51 |
| Valine |  |  |  |  |  |  |  |  |
| mean | 49.44 | 55.06 | 58.34 | 85.75 | 70.52 | 87.16 | 94.19 | 92.31 |
| SD | 14.80 | 10.99 | 17.62 | 7.38 | 26.03 | 27.42 | 10.40 | 13.39 |
| **Sugars** |  |  |  |  |  |  |  |  |
| α-D-Glucose |  |  |  |  |  |  |  |  |
| mean | 4493.91 | 4266.55 | 4317.35 | 4447.79 | 3446.10 | 3279.27 | 2401.89 | 1891.32 |
| SD | 685.64 | 694.80 | 1302.40 | 421.22 | 1087.34 | 997.68 | 1979.30 | 1664.51 |
| β-D-Glucose |  |  |  |  |  |  |  |  |
| Mean | 6993.09 | 6465.58 | 6266.69 | 6626.28 | 5203.02 | 4897.11 | 3645.00 | 2733.75 |
| SD | 1143.96 | 948.68 | 1723.83 | 590.56 | 1604.05 | 1514.98 | 3113.00 | 2540.08 |
| D-Fructose |  |  |  |  |  |  |  |  |
| mean | 2469.99 | 2287.31 | 1776.74 | 361.76 | 315.64 | 183.04 | 158.18 | 143.41 |
| SD | 451.93 | 390.67 | 499.02 | 82.74 | 118.43 | 59.35 | 16.28 | 14.78 |
| D-Mannose |  |  |  |  |  |  |  |  |
| mean | 0.00 | 0.00 | 0.00 | 0.00 | 0.00 | 0.00 | 0.00 | 0.00 |
| SD | 0.00 | 0.00 | 0.00 | 0.00 | 0.00 | 0.00 | 0.00 | 0.00 |
| **Other compounds** |  |  |  |  |  |  |  |  |
| 2,3-Butanediol |  |  |  |  |  |  |  |  |
| mean | 0.00 | 4.51 | 12.62 | 88.50 | 91.74 | 122.56 | 134.46 | 114.27 |
| SD | 0.00 | 2.55 | 9.45 | 22.59 | 21.83 | 52.88 | 39.78 | 25.97 |
| Acetaldehyde |  |  |  |  |  |  |  |  |
| mean | 0.00 | 0.00 | 0.00 | 0.00 | 0.00 | 0.00 | 0.00 | 0.00 |
| SD | 0.00 | 0.00 | 0.00 | 0.00 | 0.00 | 0.00 | 0.00 | 0.00 |
| DMSO |  |  |  |  |  |  |  |  |
| mean | 18.13 | 17.81 | 12.66 | 14.38 | 6.09 | 4.69 | 5.78 | 5.16 |
| SD | 1.02 | 7.11 | 7.66 | 3.93 | 2.43 | 1.46 | 0.70 | 0.89 |
| D-Mannitol |  |  |  |  |  |  |  |  |
| mean | 0.00 | 0.00 | 0.00 | 9009.76 | 7162.92 | 8279.26 | 9361.35 | 8179.80 |
| SD | 0.00 | 0.00 | 0.00 | 865.01 | 2292.56 | 2182.12 | 668.02 | 1006.09 |
| dUMP |  |  |  |  |  |  |  |  |
| mean | 0.00 | 374.13 | 449.95 | 217.58 | 191.69 | 235.45 | 281.06 | 255.79 |
| SD | 0.00 | 88.21 | 95.26 | 20.16 | 64.11 | 64.37 | 37.58 | 34.59 |
| Ethanol |  |  |  |  |  |  |  |  |
| mean | 385.42 | 627.27 | 761.60 | 1103.45 | 980.41 | 2057.37 | 4534.17 | 4209.72 |
| SD | 107.59 | 170.53 | 226.04 | 126.81 | 308.06 | 1107.45 | 3808.75 | 3389.78 |
| GABA |  |  |  |  |  |  |  |  |
| mean | 74.25 | 83.32 | 43.31 | 0.00 | 0.00 | 0.00 | 0.00 | 0.00 |
| SD | 14.27 | 25.47 | 43.24 | 0.00 | 0.00 | 0.00 | 0.00 | 0.00 |
| Methanol |  |  |  |  |  |  |  |  |
| mean | 29.28 | 30.69 | 36.65 | 56.84 | 56.13 | 72.47 | 95.54 | 91.95 |
| SD | 3.78 | 7.22 | 11.81 | 4.65 | 17.88 | 20.05 | 9.07 | 8.00 |
| Putrescine |  |  |  |  |  |  |  |  |
| mean | 0.00 | 0.00 | 0.00 | 0.00 | 0.00 | 83.57 | 110.36 | 65.58 |
| SD | 0.00 | 0.00 | 0.00 | 0.00 | 0.00 | 52.52 | 68.11 | 51.70 |
| Succinamide |  |  |  |  |  |  |  |  |
| mean | 23.70 | 15.80 | 64.61 | 36.72 | 44.62 | 16.50 | 19.52 | 22.31 |
| SD | 21.20 | 3.45 | 49.88 | 73.66 | 56.60 | 10.69 | 4.89 | 7.77 |
| Succinimide |  |  |  |  |  |  |  |  |
| mean | 5.15 | 5.15 | 6.94 | 16.65 | 7.13 | 4.16 | 4.76 | 4.36 |
| SD | 1.29 | 3.01 | 4.32 | 3.60 | 1.63 | 1.91 | 0.83 | 0.89 |
| Trimethylamine N-oxide |  |  |  |  |  |  |  |  |
| mean | 47.31 | 44.46 | 49.42 | 60.38 | 47.46 | 47.91 | 54.37 | 46.86 |
| SD | 9.33 | 8.88 | 14.15 | 5.64 | 15.78 | 13.94 | 5.66 | 4.33 |
| Uracil |  |  |  |  |  |  |  |  |
| mean | 1.35 | 3.81 | 10.09 | 14.57 | 12.78 | 13.67 | 16.59 | 14.35 |
| SD | 1.46 | 1.28 | 3.07 | 2.10 | 4.53 | 3.49 | 0.94 | 2.43 |
