# Supplementary material for: Microbial and metabolic characterization of organic artisanal sauerkraut fermentation and study of gut health-promoting properties of sauerkraut brine
Source: Front Microbiol. 2022 Oct 13;13:929738. doi: 10.3389/fmicb.2022.929738 (PMC9606823; doi:10.3389/fmicb.2022.929738)
Supplement: Supplementary Table S2 — P-values of statistically significant differences in metabolite concentration in sauerkraut brine from SK1 (A) and from SK2 (B) after Kruskal–Wallis test, followed by the post-hoc Dunn's test with Benjamini–Hochberg false discovery rate (FDR) p-value correction. [file Table_2.docx]

**Table S2**. p-values of statistically significant differences in metabolites concentration in sauerkraut brine from SK1 (A) and from SK2 (B) after Kruskal–Wallis test, followed by the post-hoc Dunn’s test with Benjamini–Hochberg false discovery rate (FDR) p value correction.

| **A. SK1** | **Comparison** | **p-value** |
| --- | --- | --- |
| **Metabolites** |  |  |
| Acetic acid | day 1 vs day 14  day 1 vs day 21  day 1 vs day 28  day 1 vs day 35 | 0.029  0.013  0.047  0.001 |
| D-Fructose | day 1 vs day 7  day 1 vs day 14  day 1 vs day 28 | 0.001  0.033  0.0.14 |
| D-Mannitol | day 1 vs day 14  day 1 vs day 21  day 1 vs day 35 | 0.041  0.023  0.002 |
| Lactic acid | day 1 vs day 21  day 1 vs day 35 | 0.006  < 0.001 |
| **B. SK2** |  |  |
| **Metabolites** |  |  |
| Acetic acid | day 1 vs day 21  day 1 vs day 28  day 1 vs day 35 | 0.023  < 0.001  0.001 |
| Butyric acid | day 1 vs day 35  day 2 vs day 35  day 3 vs day 35  day 7 vs day 35  day 14 vs day 35  day 21 vs day 35  day 28 vs day 35 | < 0.001  < 0.001  < 0.001  < 0.001  < 0.001  < 0.001  < 0.001 |
| D-Fructose | day 1 vs day 21  day 1 vs day 28  day 1 vs day 35 | 0.033  0.006  0.001 |
| D-Mannitol | day 1 vs day 7  day 1 vs day 28  day 2 vs day 7  day 2 vs day 28  day 3 vs day 7  day 3 vs day 28 | 0.047  0.016  0.047  0.016  0.047  0.016 |
| Lactic acid | day 1 vs day 28  day 2 vs day 28 | 0.003  0.009 |
| Propionic acid | day 1 vs day 28  day 1 vs day 35  day 2 vs day 28  day 2 vs day 35 | 0.014  0.018  0.006  0.008 |
| Succinate | day 7 vs day 28  day 7 vs day 35 | 0.003  0.001 |
